# Biochemical Analysis of Recombinant Pea Seed Coat-Specific Polyphenol Oxidase (*Pea*PPO) in Relation to Various Phenolic Substrates

**DOI:** 10.1021/acs.jafc.5c01839

**Published:** 2025-08-21

**Authors:** Adéla Franková, Matthias Pretzler, Jana Balarynová, Jana Sekaninová, Petra Krejčí, Petr Bednář, Sanja Ćavar Zeljković, Vladan Doupovec, Mária Škrabišová, René Lenobel, Marek Petřivalský, Annette Rompel, Petr Smýkal

**Affiliations:** 1 Department of Botany, Faculty of Sciences, 48207 Palacký University Olomouc, Olomouc 77900, Czech Republic; 2 Fakultät für Chemie, Institut für Biophysikalische Chemie, 27258 Universität Wien, Wien 1090, Austria; 3 Department of Biochemistry, Faculty of Sciences, 48207 Palacký University Olomouc, Olomouc 77900, Czech Republic; 4 Department of Analytical Chemistry, Faculty of Sciences, Palacký University, Olomouc 77900, Czech Republic; 5 Czech Agrifood Research Center, Olomouc 77900, Czech Republic; 6 Czech Advanced Technology and Research Institute (CATRIN), Palacký University, Olomouc 77900, Czech Republic; 7 Laboratory of Growth Regulators, Faculty of Sciences, 48207 Palacký University Olomouc, Olomouc 77900, Czech Republic

**Keywords:** legumes, pea, phenolics, polyphenol oxidase, seeds, tyrosinase

## Abstract

The seed coat serves as the primary protective barrier, offering mechanical and chemical defense for the embryo. It contains various metabolites, including phenolic compounds, which can be oxidized by polyphenol oxidase (PPO) to form oligomers. In this study, we heterologously expressed a 515 amino acid protein derived from wild pea (*Pisum elatius*), omitting its N-terminal signal sequence, and analyzed its biochemical properties. The recombinant *Pea*PPO required sodium dodecyl sulfate (SDS) for activation and exhibited activity between pHs 5.2 and 7.0, peaking at pH 6.0 with 0.25 mM SDS. Tropolone and its isomer thujaplicin were the most effective inhibitors. *Pea*PPO catalyzed reactions with seed coat-derived substrates, displaying activity toward phenols, catechols, and pyrogallols, with the highest affinity for catechols. Principal component analysis of LC-MS/MS-derived phenolic profiles demonstrated that PPO+ and ppo– genotypes differ significantly in their accumulation of PPO substrates and inhibitors. These findings confirm that *Pea*PPO possesses both monophenolase and catechol oxidase activities, identifying it as a tyrosinase.

## Graphical abstract

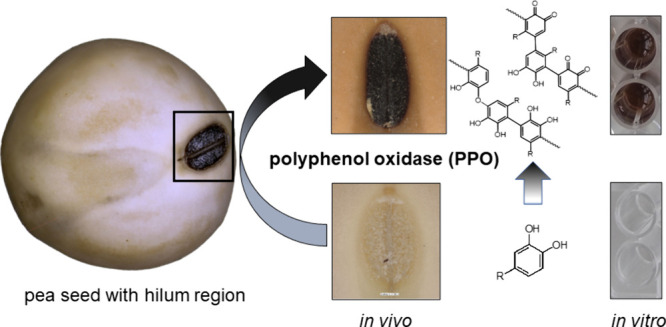

## Introduction

The seed coat is the main protective layer of the seeds, providing mechanical and chemical protection to the vulnerable embryo. It contains many different metabolites, including phenolic substances. Several types of polyphenols can be found in the seed coat, and the main groups are flavonoids, lignins, and lignans.^1,2^ Legume seed coat development and gene expression have been studied, and numerous genes involved in flavonoid, phenylpropanoid, and flavone biosynthesis have been identified in several legumes^3–5^ including pea.^6–10^ Plant polyphenols are of interest due to both negative and positive effects on the nutritional aspects of our food as well as protective properties to the plants.^11^

One of the differentially expressed genes between wild and domesticated pea seeds encodes polyphenol oxidase (PPO).^12^ PPOs (catechol oxidases, EC 1.10.3.1; tyrosinases, EC 1.14.18.1; and aureusidin synthase, EC 1.21.3.6) are a group of type-III-copper-containing enzymes together with laccases (EC 1.10.3.2) and hemocyanins.^13,14^ PPOs are a highly diverse group of enzymes that, except for the highly conserved central part, differ widely in amino acid sequence, function, temporal and spatial expression, and substrate specificity.^14–17^ Several *PPO* genes are seed-specific and involved in seed coloration.^12,18–20^ Diversity in gene number, sequence, tissue specificity, and substrate specificity across plant species suggest that the PPOs have long-term roles in fitness and adaptation to environmental factors.^21^

PPOs catalyze the oxidation of phenolic compounds into highly reactive quinones.^22^ Polymerization of quinones causes the postharvest browning of cut or processed plant tissues,^23^ but the native physiological functions of PPOs in undamaged, intact plant cells are not well understood. PPO is proposed to be involved in plant defense,^24,25^ reactive oxygen species (ROS) metabolism, and the biosynthesis of substances required for protection.^26^ Its expression was found to be increased during biotic^27,28^ and abiotic stresses. PPO plays an important role in the biosynthesis of the pigments (aurones, betalains) and lignin in phenylpropanoid and tyrosine metabolism.^16^

The loss-of-function of seed-expressed *PPO* genes has been found in several domesticated crops, such as foxtail millet,^29^ rice,^30^ barley,^31^ and pea.^12^ This loss of function is associated with a domestication status, yet it is not a prerequisite for it.^12,30,31^ The possible explanations of PPO selection include direct selection due to the presence of antinutritional compounds affecting digestion, palatability, or the result of cultural preference for nonbrowning food. Postharvest browning of fruits and vegetables causes significant economic and commercial losses due to the deterioration of taste, appearance, and aroma as well as reduced nutritional quality.

In plants, PPOs are perhaps best known for their role in postharvest browning: secondary reactions of PPO-generated *o*-quinones with cellular nucleophiles leading to the familiar coloration of plant products after mechanical damage by herbivores or crop harvest. However, in certain cases, this can be useful, for example, in the fermentation process in tea production or preservation of proteins in forage crops.^16^ Due to this browning effect, PPO enzymes were analyzed in fungi, insects, animals, and various plants.^17,32^ The processes of enzymatic browning are due to the activity of polyphenol oxidase (PPO) and peroxidase (POX, EC 1.11.1.7) enzymes, which catalyze the oxidation of phenolics leading to the formation of quinone compounds. Afterward, the quinone compounds undergo a nonenzymatic polymerization process that leads to the production of dark melanin pigments^33^ or other polymers.^26^ This pigmentation is visible in the seed coat,^34^ and also pea and fava bean hilum, and it relates to PPO activity.^12,23^ While the initial steps of phenylpropanoid biosynthesis are relatively well understood, the terminal parts, especially the final oxidation and polymerization steps, are far less understood.^35^ A large spectrum of flavonoids and phenolic acids can act as substrates^18,23,35,36^ and thereby serve as precursors for melanin structures;^37^ however, the natural substrates are largely unknown.^38^ *In vitro* tested substrates involve chlorogenic and caffeic acids, catechin, or phloretin.^39^ We have shown that these phenolic compounds accumulate during the pea seed coat development,^7,10,12^ but their accumulation can occur as a result of the stress response, particularly in defense against pathogens.^15,24,40,41^ Many PPOs cooperate with POXs and have diverse and overlapping physiological functions in plants, which include involvement in redox metabolism, responses to wound healing, defense against pathogens or insects, synthesis of lignin and suberin, and cross-linking of cell wall components.^24,26^

Using genetic, transcriptomic, proteomic, and metabolomic approaches, we previously identified a polyphenol oxidase (*PPO*) gene in the pea seed coat. We have shown that the functionality of the *PPO* gene relates to the oxidation and polymerization of phenolic compounds in the seed coat.^12^ Additionally, imaging mass spectrometry supported the hypothesis that hilum pigmentation is dependent on the presence of both phenolic precursors and sufficient PPO activity. In this study, we have heterologously expressed and studied the biochemical properties of PPO cloned from wild pea (*Pisum elatius*) seed coat.

## Materials and Methods

### Plant Material

As representatives of cultivated modern pea (*Pisum sativum* L.) cv. Trendy, primitive domesticated landrace JI92 (with nonfunctional *ppo* gene) and wild pea (*P. elatius* M.Bieb.) JI64 and JI1794 (with functional *PPO* gene) were chosen based on the previous studies.^7,10,12^ The plants were cultivated, and seeds were stored as described previously.^10,12^

### Bioinformatic Analysis of *Pea*PPO

Currently, available pea genomes contain two *PPO* genes^42,43^ but only one *PPO* gene is seed-specific and linked to the hilum and seed coat pigmentation phenotype.^12,44^ The protein sequence of wild pea *P. elatius* JI64 ^12^ was retrieved from GenBank USF91943.1 and blasted against the NCBI BlastP protein sequence database. Plant protein sequences with an identity higher than 70% were analyzed for conserved amino acids by WebLogo.^45^ AlphaFold^46^ and Phyre2 ^47^ were used to predict the 3D structure of *Pea*PPO. Multiple sequence alignment was constructed using Clustal Omega within SnapGene 7.2 software (www.snapgene.com). The predicted 3D structure of *Pea*PPO was aligned with the crystal structure of the PPO with the highest sequence identity using PyMOL (https://www.pymol.org). The side chains of the conserved His residues, as well as the *Pea*PPO-specific His residue, were highlighted, and the positions of the two copper atoms were modeled based on the aligned crystal structure.

### Isolation and Expression of the Gene Encoding PeaPPO

Although the *Psat1g206360* gene does not contain introns, we used RNA as the source to amplify the respective *PPO* gene. The full-length coding region of the pea encoding *PPO-1* gene (*Psat1g206360*) was amplified from total RNA isolated from the seed coat of wild pea JI64 ^12^ using specific primers (*Pea*PPO_*Eco*RI_fwd and *Pea*PPO_XhoI_rev, Table S1), cloned into pGEM-T vector, and transformed into chemically competent *Escherichia coli* TOP10 cells (Thermo Fisher Scientific). Upon analysis by Sanger sequencing, a verified clone was chosen for subcloning into the pGEX-6P-SG expression vector.^48^

### Heterologous Expression and Purification of the Recombinant PeaPPO Protein

The gene for *Pea*PPO without its signal sequence consists of 1548 bp encoding 515 amino acids, which corresponds to a 57.89 kDa protein starting with the amino acid sequence SPISPPDL. The respective part of the gene was amplified from the selected pGEM-T clone by PCR using the primer pair l*Pea*PPO_Esp3I_fwd and *Pea*PPO_Esp3I_rev (Table S1) with the Q5 DNA polymerase (New England Biolabs) according to the manufacturer’s recommendations. The pea *PPO* gene was N-terminally fused with the glutathione S-transferase (GST)-tag of the pGEX-6P-SG vector. The human rhinovirus 3C protease (HRV3C) recognition sequence (LEVLFQ|GP) was located between GST and PPO enabling the controlled proteolytic dissociation of the two proteins. The two fused genes (GST-*Pea*PPO) were efficiently overexpressed in *E. coli* BL21 (DE3) using the synthetic tac promoter of the pGEX-6P-SG vector.^48,49^ The expression batches were inoculated with saturated overnight cultures and grown at 37 °C under shaking for 4 h until the OD_600_ reached a value between 0.6 and 0.8 when the expression was started by adding 0.5 mM isopropyl β-d-1-thiogalactopyranoside (IPTG) and 0.5 mM CuSO_4_. Afterward, the expression cultures remained at 16 °C under shaking for 65 h. The culture was then collected by centrifugation at 8000×*g* for 30 min at 4 °C. Lysis of the cells was done by a freeze–thaw technique using liquid nitrogen: the pellets were resuspended in 35 mL of lysis buffer (50 mM Tris-HCl pH 7.5, 200 mM NaCl, 1 mM EDTA, and 50 mM sucrose). Lysozyme (0.5 g/L) and protease inhibitors (1 mM PMSF and 1 mM benzamidine) were added, and the resulting suspensions were incubated for 45 min under shaking on ice. After this initial incubation, the solution was frozen five times in liquid nitrogen and thawed in water at room temperature. After the third thawing, DNase solution (final concentrations: 10 mg·L^–1^ DNase I, 3 mM MgCl_2_, and 10 mM CaCl_2_) was added. Finally, the lysate was centrifuged for 60 min at 4000×*g* and 4 °C and filtrated through a 0.45 μm PES membrane.

### Purification of the Recombinant PeaPPO Protein

The chromatographic purifications were carried out using an Äkta Purifier (GE Healthcare) placed in a refrigerator at 4 °C. All purification steps were carried out at 4 °C. The filtrated lysates were injected using a sample pump and applied onto a prepacked 5 mL GSTrap FF column (GE Healthcare) using 50 mM Tris-HCl pH 7.5 and 200 mM NaCl as the binding buffer. The target proteins were eluted with 50 mM Tris-HCl pH 7.5, 200 mM NaCl, and 15 mM reduced glutathione. Fractions containing the GST-fusion protein were pooled and concentrated using a Vivaspin ultrafiltration device with a 30 kDa molecular weight cutoff. The buffer was exchanged to 50 mM Tris-HCl pH 7.0, 200 mM NaCl, and 1 mM EDTA using the same ultrafiltration device, and the samples were mixed with the GST-HRV3C protease^50^ at a 1:50 mass ratio (protease:fusion protein). The proteolysis was carried out for 48 h at 4 °C. Subsequently, the noncleaved protein and the added protease were trapped by the same column used for the initial protein capture while *Pea*PPO was eluted in the flowthrough (Figure 1). The buffers used in the second round of affinity chromatography had the same recipes as those used in the first round of chromatography. The protein fractions were analyzed by SDS-PAGE, and the protein bands were stained with Coomassie brilliant blue CBB-G250 in the presence of Al^3+^.^51^

**Figure 1:**
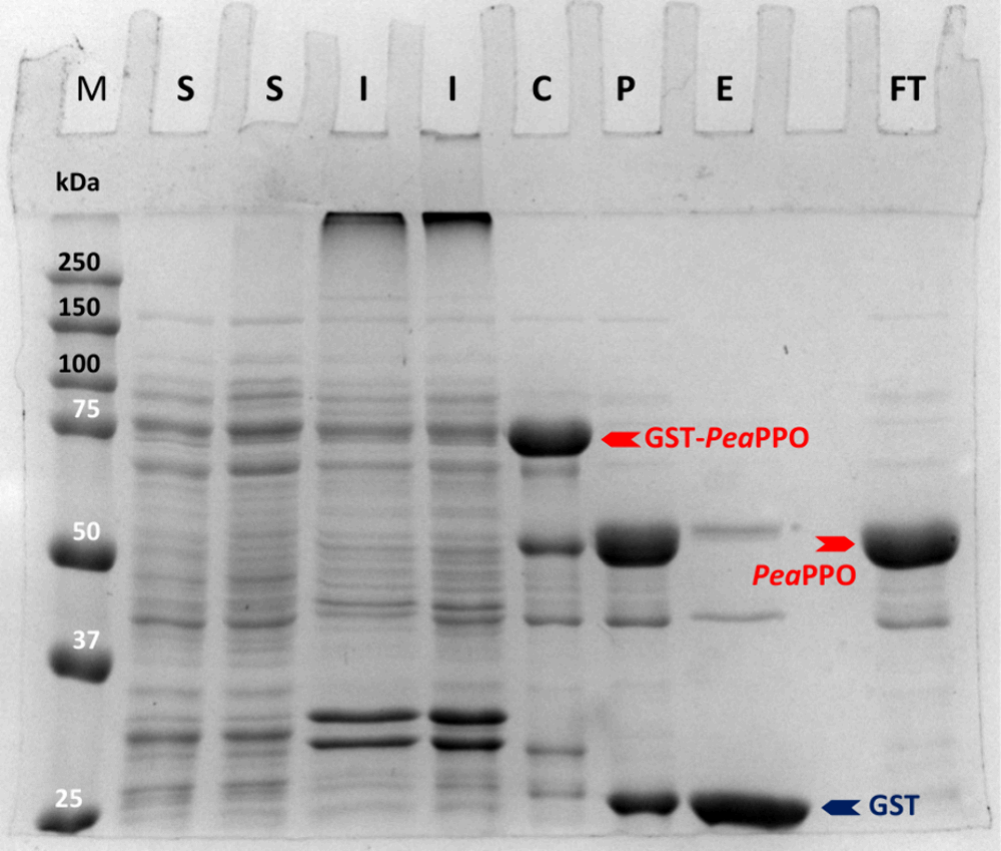
SDS-PAGE showing the purification stages of *Pea*PPO. Samples were reduced, heat denatured, and separated on a 10%(m/v) acrylamide gel. An amount of 60 μg of total protein was loaded for the lysate fractions (S and I), while 20 μg of proteins of each chromatographic fraction (C, P, E, and FT) was applied. The protein bands were stained with Coomassie brilliant blue CBB-G250 in the presence of Al^3+^. M: molecular weight marker (protein weight in kDa), S: soluble fraction of the cell lysate (two batches), I: insoluble fraction of the cell lysate (two batches), C: eluate of the first affinity chromatography, P: eluate of the first affinity chromatography incubated with the protease GST-HRV3C, E: eluate of the second affinity chromatography, FT: flowthrough of the second affinity chromatography, GST-*Pea*PPO: fusion protein of glutathione S-transferase from *Schistosoma japonicum* (GST) and *Pea*PPO (84.6 kDa), *Pea*PPO: purified *Pea*PPO after proteolytic removal of the GST tag (58.2 kDa, contains three vector-derived amino acids at its N-terminus: glycine-proline-methionine), GST: cleaved-off GST tag (26.4 kDa).

### Identification of PeaPPO by Proteomic Analysis

A sample of *Pea*PPO (5 μL containing 19.6 μg of enzyme) was diluted with 45 μL of 50 mM TEAB buffer (pH 8.0) supplemented with 1% (w/v) sodium deoxycholate, 10 mM NaCl, and 1 mM DTT.^52,53^ The mixture was shaken for 10 min at 56 °C and 1000 rpm in a thermomixer. Afterward, 2.5 μL of 200 mM DTT was added to the mixture and incubated in a thermomixer for 45 min at 56 °C and 500 rpm. Alkylation of free cysteines was done by adding 10 μL of 250 mM iodoacetamide solution. The mixture was incubated and shaken for 45 min at room temperature and 500 rpm in the dark. Finally, unreacted iodoacetamide was quenched by adding 2.5 μL of 200 mM DTT for 15 min at RT and mixed at 500 rpm. Aliquots of the resulting *Pea*PPO solution (10 μL) were mixed with 40 μL of 50 mM TEAB (pH 8.0), and 1 μL of SoluTrypsin was added. In-solution digestion was done at 37 °C overnight in the dark. The next day, the mixture was acidified with 98% (v/v) formic acid up to 5% (v/v) to stop digestion and precipitate sodium deoxycholate. Precipitation was performed for 5 min at 25 °C and 1000 rpm in a thermomixer, followed by centrifugation for 5 min at 13,400×*g*. The supernatant was transferred to a new tube and diluted with 40 μL of 5% (v/v) FA, and peptides were purified using the Stage-Tip procedure^54^ with a reverse phase (C18). Purified peptides were evaporated in a vacuum concentrator and redissolved in 50 μL of 5% (v/v) formic acid. An aliquot was analyzed by nLC-MS/MS analysis (Tims TOF Pro2, Bruker Daltonics) using the settings from ref 55. The collected Tims-MSMS data were processed and searched using fragPIPE software, version 22.0.^56^ with the MSFragger engine.^57^ Peptide and protein identification was performed against the database containing the reference proteome of *P. sativum* downloaded from the UniProt repository (UP001058974_2024_07_25) and supplemented with the sequence of the recombinant *Pea*PPO protein, common contaminants, and reversed sequences. The peptide sequences were validated using the Target-Decoy PSM approach.

### Protein Concentration

The protein concentration was determined spectrophotometrically via the absorbance at 280 nm of an appropriately diluted (0.2 cm^–1^ < A_280_ < 1 cm^–1^) enzyme solution. The molar absorption coefficient was calculated from the number of tryptophan (8), tyrosine (16), and cystine residues (2) in the primary sequence of *Pea*PPO.^58^ For this calculations, the presence of two disulfide bridges that are structurally conserved in plant PPOs^59,60^ was assumed for *Pea*PPO, resulting in a molar decadic absorption coefficient of ε_280_ = 68,090 M^–1^ cm^–1^.

### Determination of Copper Content

To determine the content of copper ions in the enzyme, the protocol of Hanna et al., (1988)^61^ was used. Briefly, 100 μg of enzyme (25 μL solution) was mixed with 15 μL of 0.5 M sodium phosphate buffer pH 6. An amount of 20 μL of 1 M sodium ascorbate and 90 μL of 0.5 g·L^–1^ 2,2′-bichinoline in glacial acetic acid were added. The protein is denatured due to the low pH, which releases the copper ions bound at the active site. Cu­(II) ions are reduced to Cu­(I) by the ascorbate in the solution, and the complexation of Cu­(I) ions by 2,2′-bichinoline produces a pink product, which was measured spectrophotometrically at 546 nm after 10 min of incubation at 25 °C. The measurements were done in triplicate.

### Determination of the pH Optimum

For the determination of the optimum reaction pH, a PSG buffer^62^ containing phosphoric acid (100 mM), succinic acid (100 mM), and glycine (100 mM) was used. The pH optimum of the *Pea*PPO was studied in a pH range from 2.0 to 12.0. PSG buffer (50 mM), 0.25 mM SDS, 4 mM tyramine, and 3.9 μg of *Pea*PPO protein were used in a total reaction volume of 200 μL. Measurements were always performed in triplicates and followed at 470 nm for 2.5 h. To cover the pH range from 5.0 to 7.0 more accurately, the measurements were done at 0.2 pH intervals in that range while 1.0 pH unit intervals were used outside of it. The pH optimum obtained from this assay was used in all subsequent experiments.

### Determination of the Optimal SDS Concentration for Latent PeaPPO Activation

The optimal concentration of SDS (where *Pea*PPO activity is highest) was determined by measuring the PPO activity with tyramine as substrate in triplicate with 0.025, 0.050, 0.100, 0.250, 0.5, 0.750, 1.0, 1.5, 2.0, 3.0, and 4.0 mM SDS. The optimal SDS concentration obtained from this assay was used in all subsequent experiments.

**Table 1:** Effect of Various Inhibitors on*Pea*PPO Activity^a^

substrate	inhibitor	% inhibition
tyramine	tropolone	100 ± 0.0
	kojic acid	80 ± 7.1
	phenylthiocarbamide	52 ± 5.0
	EDTA	11 ± 2.1
	thujaplicin	83 ± 1.1
dopamine	tropolone	99 ± 0.6
	kojic acid	46 ± 5.7
	phenylthiocarbamide	98 ± 0.7
	EDTA	10 ± 2.8
	thujaplicin	99 ± 0.6

a Measured in 200 μL of 50 mM MES pH 6.0 with 0.25 mM SDS as an activator, either 1 mM dopamine or 2 mM tyramine as a substrate, 39 ng of *Pea*PPO (reactions with dopamine) or 3.9 μg of *Pea*PPO (reactions with tyramine) and 0.1 mM of the respective inhibitor. All measurements were performed in triplicate, reported is the average and standard deviation of the measured degree of inhibition calculated as 100% minus the ratio of the activity with the inhibitor and the activity in the absence of the inhibitor (2 mM tyramine: 2.3 ± 0.11 U mg^–1^, 1 mM dopamine: 79 ± 19 U mg^–1^).

### Identification of Suitable Wavelengths for the Monitoring of *Pea*PPO Activity

To find a suitable wavelength for spectrophotometric activity assays, 0.5 mM of the respective substrate was incubated with l*Ab*PPO4 (latent polyphenol oxidase number 4) from the mushroom *Agaricus bisporus* ^50^ in the respective buffer for 1 h during which UV–vis spectra (200–600 nm) of the solution were recorded every minute. In addition, chemical oxidation with five equivalents of NaIO_4_ was applied and monitored in the same way. The final measurement wavelengths (Table 2) were needed to display a sufficiently high absorbance to allow the detection of minute amounts of oxidized substrate and a stable reading over at least the longest measurement time^63^ for both the enzymatic and the chemical system. The appropriate wavelength and the molar absorption coefficients (Table 2) were determined using NaIO_4_ as the chemical oxidant.^63^ The molar absorption coefficients were determined by linear regression from absorbance–concentration curves determined with the respective substrate at the appropriate wavelengths. The molar absorption coefficient for the monophenol phloretin has already been reported in a previous study.^49^

**Table 2:** Substrate Specificity of *Pea*PPO

parameter	substrate	λ [nm]	ε [M^–1^cm^–1^]	relative activity [%] ± SD
phenols	phloretin	455	11,715^a^	21.1 ± 6.9
	tyramine	470	1240	5.3 ± 0.6
catechols	(−)-epicatechin	380	4900	58.4 ± 4.1
	4-methylcatechol	400	1570	108.6 ± 10.8
	dopamine	470	1240	100 ± 6.8
	L-DOPA	470	2405	49.6 ± 0.3
	chlorogenic acid	380	1910	41.8 ± 15.8
	caffeic acid	380	1740	18.2 ± 0.4
	2,3-dihydroxybenzoic acid	400	1155	0.4 ± 0.2
pyrogallols	pyrogallol	430	1185	59.3 ± 4.0
	myricetin	480	1055	72.3 ± 0.9
	gallocatechin gallate	420	240	75.0 ± 4.2

a Kampatsikas et al., 2019.^65^ The enzyme activity is shown as relative to the activity with dopamine (considered as 100%). The relative activity represents the mean ± standard deviation.

### PeaPPO Substrate Specificity

Several substrates, including phenols, catechols, and pyrogallols, were used to determine the substrate specificity of *Pea*PPO. The activity was determined spectrophotometrically by measuring the accumulation of the colored reaction product using a microplate reader (Synergy HT, BioTek, USA). The reaction mixture contained 200 μL of 50 mM MES (pH 6) with 0.25 mM SDS (for activation), 1 μg of *Pea*PPO (2 μL of a 1:8 dilution with 50 mM MES pH 6), and 10 μL of the respective substrate (1 mM final concentration). The substrates were dissolved in water, except for phloretin, caffeic acid, chlorogenic acid, and 2,3-dihydroxybenzoic acid, which were dissolved in ethanol. PPO activity was calculated from the slope of the steepest, linear part of the absorbance–time curves that correspond to the steady-state rate of substrate conversion. One unit of *Pea*PPO activity was defined as the amount of enzyme tha**t** catalyzes the formation of 1 μmol of reaction product (*o*-quinones in the case of PPOs) per minute at 25 °C. The relative activity is reported considering the activity in the presence of 1 mM dopamine as 100%. All measurements were done in triplicate.

### Testing of *Pea*PPO Inhibitors

The effects of several inhibitors, namely, tropolone, thujaplicin, EDTA, kojic acid, and phenylthiocarbamide, on *Pea*PPO activity were studied as previously.^64,65^ The reaction mixture contained a 0.1 mM inhibitor, 2 mM tyramine (3.9 μg of *Pea*PPO) or 1 mM dopamine (39 ng of *Pea*PPO), 50 mM MES pH 6.0, and 0.25 mM SDS. The final volume was 200 μL. The activity of *Pea*PPO was measured spectrophotometrically at 475 nm for up to 1 h at 25 °C. All measurements were done in triplicate.

### LC-MS/MS Analysis of Phenolic Metabolites in the Pea Seeds

Homogenized seed coats from mature dry seeds (≈20 mg) were mixed with 1 mL of solvent (acetone:water:acetic acid, 80:19:1, v:v:v) and sonicated for 10 min in an ultrasonic bath at room temperature. After centrifugation at 14,500×*g*, the supernatant containing free phenolics was transferred into a new vial and the solvent was evaporated to dryness under vacuum at 40 °C and redissolved in 50 μL of mobile phase (90%(v/v) 15 mM formic acid (pH 3, adjusted with NH_4_OH) and 10%(v/v) ACN) containing internal standards (salicylic acid-d_4_ and p-coumaric acid-_d6_) in the concentration of 5 μmol/L each. The pellet remaining after the supernatant removal was subjected to hydrolysis to free phenolic compounds bound with sugars, proteins, etc. NaOH (200 μL) (4 mol/L) was transferred to the tube with pellet, mixed, and sonicated at 50 °C for 10 min. For acidification, 100 μL of concentrated hydrochloric acid was added, and samples were mixed and let to cool down. Then, 200 μL of water was added and samples were mixed. After that, the free phenolic compounds were extracted with 2 × 0.5 mL of diethyl ether. Finally, the solvent was removed under a vacuum at 30 °C and the sample was redissolved in 50 μL of mobile phase containing internal standards (salicylic acid-d_4_ and *p*-coumaric acid-d_6_) at the concentration of 5 μmol/L each. The analysis of phenolic acids and flavonoids was performed according to the protocol described in our previous study.^66^ All standards and reagents were from Sigma-Aldrich Company (Prague, Czech Republic), and the measurements were performed in triplicate. Experimental results were presented in tables and graphs as the mean ± standard deviation of three independent replications. Pearson correlations were determined to observe the correlation between the levels of phenolic compounds and PPO activity at the level of significance *p* < 0.05. Heatmaps and correlation analysis were performed in RStudio (2023.12.0, Posit Software, PBC, Boston, MA, USA) using the *gplots* and *corrplot* packages.

### UHPLC-MS Analysis of the Products of *Pea*PPO Reaction

The products of the reactions were analyzed using UHPLC separation (ACQUITY, Waters) with UV (PDA eLambda detector, Waters) and MS detection (Select Series Cyclic IMS, Waters) in negative ionization mode. Mobile phase A consisted of water with 0.1% formic acid, and mobile phase B consisted of methanol with 0.1% formic acid (both v/v). The flow rate of the mobile phase was set to 0.150 mL/min, the time of analysis was 20 min, and the parameters of linear gradient elution were as follows: initially, 0.1% of mobile phase B linearly ramped up to 100% B over 15.00 min. Reaction mixtures were diluted (1:10) in a mixture of the mobile phases (A:B, v:v, 1:1), and their separation was performed using a ZORBAX Eclipse Plus C18 column (Agilent) thermostated at 30 °C. PDA detector wavelength was set in the UV region of 220–420 nm. Parameters of the mass spectrometer were: spray voltage 2.5 kV, cone voltage 25 V, desolvation gas (>99.99% N_2_) flow 600 L/h, and desolvation gas temperature of 220 °C.

## Results

### Bioinformatic Analysis of *Pea*PPO

Protein sequences that were previously biochemically characterized as PPOs with predominant tyrosinase or catechol oxidase activity were compared with *Pea*PPO. The highly conserved regions of the two Cu-binding domains were identified, with the *Pea*PPO-specific His213 residue located in a conserved region close to the CuA binding domain (Figure 2). WebLogo analysis revealed that the frequency of this His residue in plant proteins with an identity to *Pea*PPO on the amino acid level above 70% is 0.328 in contrast to the typically occurring Tyr residue (Figure 2B). This substitution potentially increases the hydrophilicity in an otherwise very hydrophobic part of the protein, potentially widening the active site pocket (Figure 2C,D). The full-length functional pea *PPO* is encoded by an 1809 nt long gene without an intron, encoding 602 amino acids yielding a 67.4 kDa protein with a calculated pI of 6.66. In our study, we produced a 515 amino acid long protein obtained by removing the two N-terminal signal sequences (87 amino acids, 9.52 kDa).

**Figure 2:**
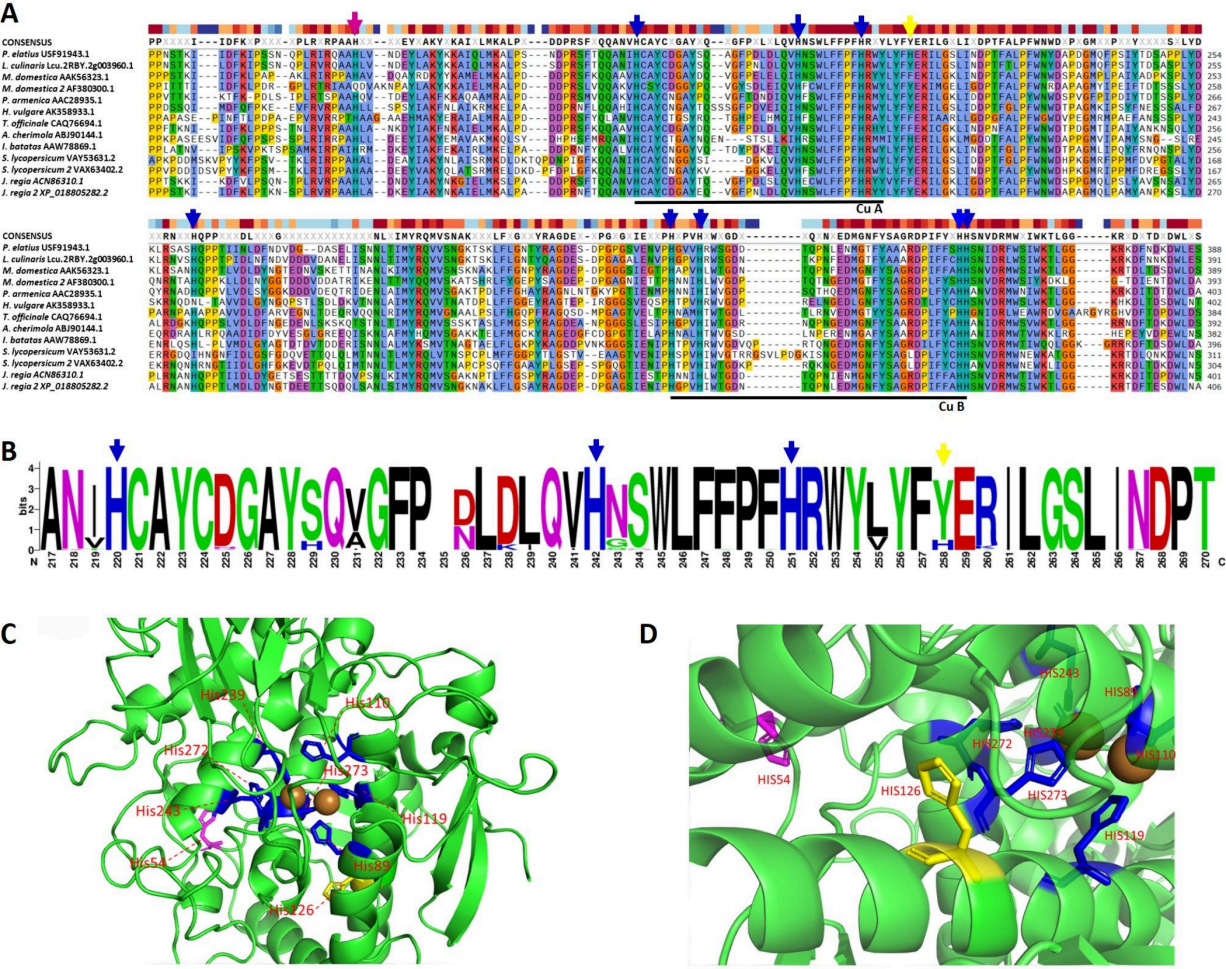
Bioinformatic analysis of *Pea*PPO. Sections of a multiple protein alignment (Clustal Omega) of biochemically characterized PPOs with confirmed tyrosinase or catechol oxidase activity. Black lines delineate the known copper-binding domains, while arrows indicate the His residues that coordinate copper atom binding. The yellow arrow highlights a *Pea*PPO-specific His residue, and the magenta arrow points to a conserved residue outside of the copper-binding domains (A). WebLogo representation of the CuA binding domain from *Pea*PPO’s top identity proteins, showing conserved His residues (blue arrow) and the additional *Pea*PPO-specific His residue (yellow arrow) (B). Predicted 3D structure of *Pea*PPO displaying two copper atoms coordinated by the conserved His residues (blue), the *Pea*PPO-specific His residue (yellow), and a distant conserved His residue (magenta)­(C). Close-up view of the additional *Pea*PPO-specific His residue (yellow) (D).

### *Pea*PPO Protein Identification and Sequence Confirmation by Proteomic Analysis

The identity of purified *Pea*PPO after the removal of the GST tag was confirmed by digestion with trypsin and subsequent proteomic analysis. Recombinant *Pea*PPO has 515 amino acids, and 27 unique peptides were identified (Figure 3, Figure S1, and Table S2), which confirm 64.3% of the sequence. Searching the same list of identified peptides against the reference proteome of *P. sativum* from the UniProt repository, an enzyme tyrosinase copper-binding domain-containing protein from pea (A0A9D5H103_PEA) was identified as the best match. Performing CLUSTAL multiple sequence alignment of recombinant *Pea*PPO and A0A9D5H103_PEA confirmed 98.83% identity (509 of 515 amino acids are identical) of both proteins (Figure 3). This is well within the expected variation range for plant PPOs isolated from different individuals of the same species.

**Figure 3:**
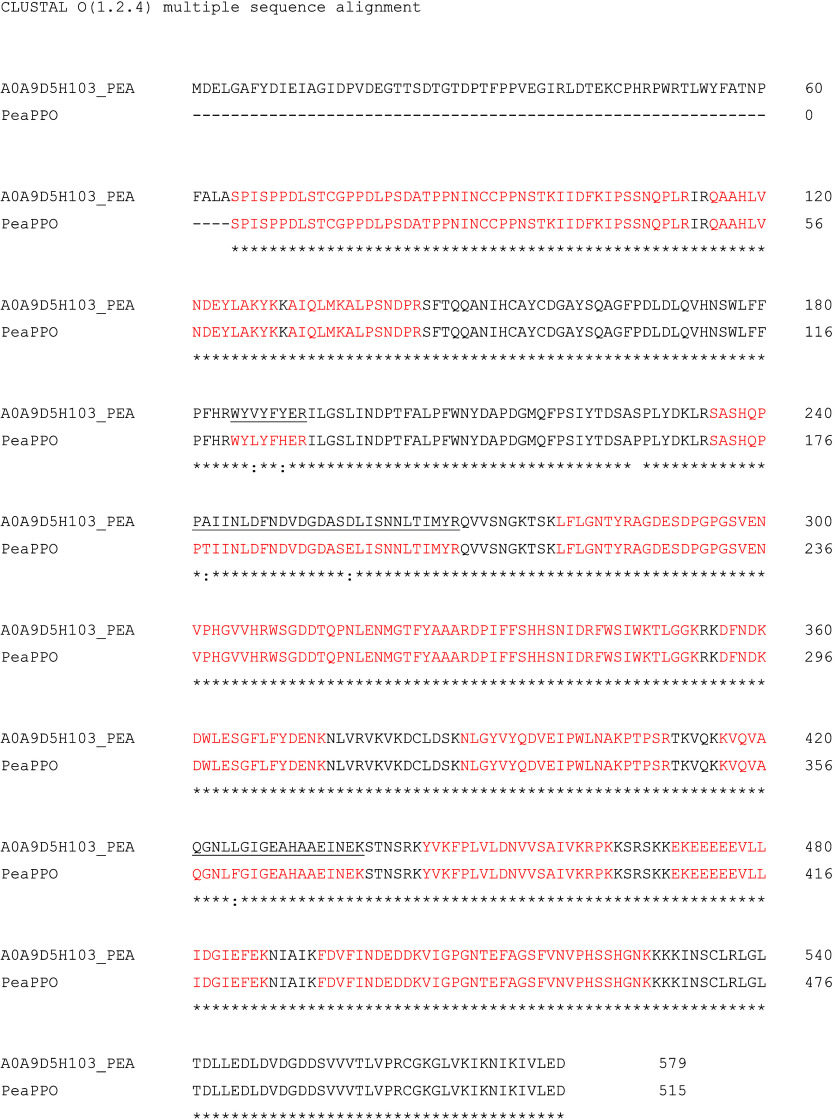
Sequence alignment of the tyrosinase copper-binding domain-containing protein (A0A9D5H103_PEA) and recombinant *Pea*PPO performed by CLUSTAL O (1.2.4) using a web tool on the UniProt.org (align tool). Peptide sequences identified in both sequences using the proteomic analysis are highlighted in red. Black lines underline peptide sequences identified only in the recombinant *PeaPPO*.

### Characterization of the *Pea*PPO Protein

The pH optimum of *Pea*PPO was analyzed in the pH range from 2.0 to 12.0. *Pea*PPO was active in the pH range from 5.2 (*A*_spec_ 0.11 ± 0.028 U mg^–1^) to 10.0 (*A*_spec_ 0.29 ± 0.062 U mg^–1^) with the highest activity at pH 6.0 (*A*_spec_ 1.88 ± 0.095 U.mg^–1^) (Figure 4A). The quantification of copper ion content of *Pea*PPO based on spectrophotometrical measurement of the Cu^I^-2′-biquinoline complex at 546 nm revealed that the recombinant *Pea*PPO protein contained approximately 3.2 ± 0.2 copper ions per protein chain. Detergents such as SDS are usually used to convert the PPO to the active form. The recombinant *Pea*PPO was not active in the absence of SDS in the reaction mixture. The maximum activity of *Pea*PPO was recorded using 0.25 mM SDS (*A*_spec_ 6.6 ± 0.15 U mg^–1^). Higher SDS concentrations slightly decreased *Pea*PPO activity (Figure 4B).

**Figure 4:**
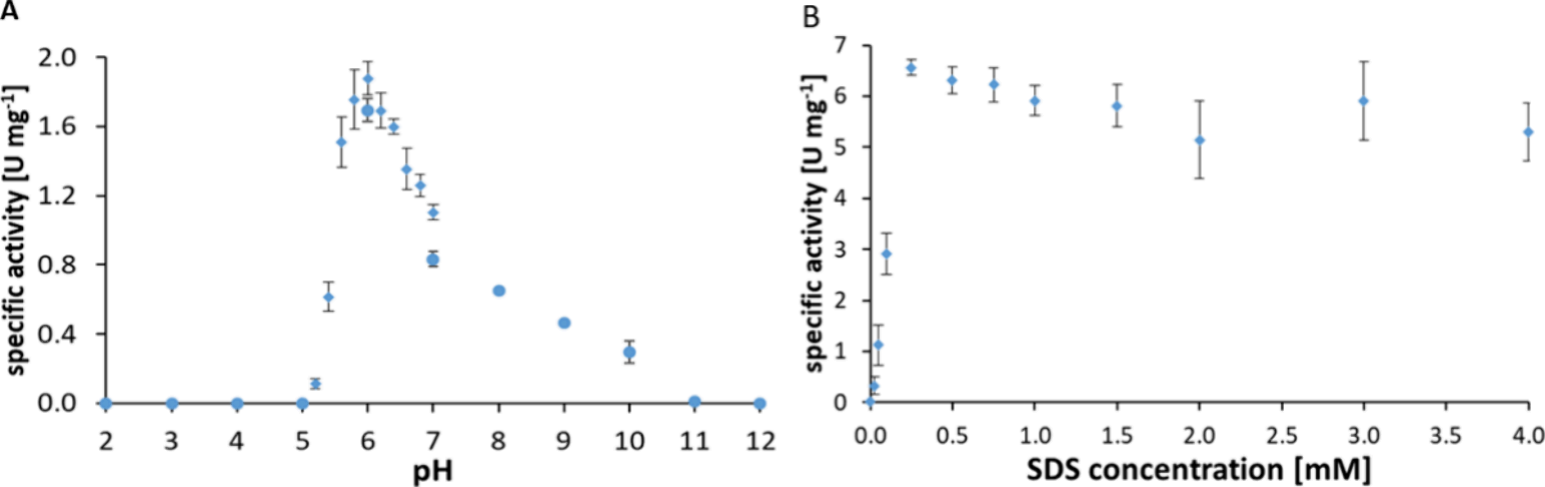
Effect of pH and SDS on the activity of *Pea*PPO. The enzyme activity was determined in 50 mM PSG buffer using 4 mM tyramine as a substrate and 0.25 mM SDS for activation of the latent enzyme (A). The effect of different SDS concentrations on *Pea*PPO activity. The enzyme activity was determined using 4 mM tyramine in 50 mM MES pH 6.0 (B). Data points represent the average of three activity measurements; the bars show mean ± standard deviation.

### Effect of Inhibitors on *Pea*PPO Activity

PPO activity can be inhibited by applying metal ion chelators (such as EDTA) or compounds structurally resembling the phenolic substrates (such as tropolone, which competes with substrate binding to the copper ions at the active site). The effect of various inhibitors on *Pea*PPO activity was tested (Table 1). Tropolone and its isomer thujaplicin were the most effective inhibitors of *Pea*PPO activity.

### Analysis of Endogenous Candidate Substrates from Pea Seeds

In addition to untargeted metabolomic profiling,^10^ we have used the targeted LC-MS/MS method to quantify selected phenolic and flavonoid metabolites present in the mature seed coats of selected pea genotypes. These represented both wild peas with functional *PPO* and pigmented seeds (JI64 and JI1794), as well as landrace (JI92) with pigmented seeds and a nonfunctional *PPO* gene and a modern pea variety (cv. Trendy) with both nonpigmented seed coats, and a nonfunctional *PPO* gene. Among measured metabolites, several represent previously described PPO substrates (gallic acid, caffeic acid, gallocatechin, epigallocatechin, and phloretin), all being present in high concentrations in the seeds coat, except for cv. Trendy, which had very low amounts of all measured metabolites (Table S3). Both wild genotypes contained significant amounts of epigallocatechin in its free form, especially JI64, while the second most abundant metabolites were flavonoid luteolin and 2,3-dihydroxybenzoic acid. All these compounds contain *ortho*-dihydroxy groups (i.e., they are catechols), which makes them suitable substrates of PPO. Landrace JI92 also contained significant levels of both luteolin and 2,3-dihydroxybenzoic acid, at higher levels than wild genotype JI1794, but the levels of epigallocatechin were significantly lower compared to the wild genotypes. In addition, modern cv. Trendy also contained the least amounts of all metabolites quantified, generally 2 orders of magnitude lower compared to others (Table S3).

To investigate the relationship between phenolic composition and PPO status, four principal component analyses (PCA) were conducted (Figure 5 and Figure S4). The PCA plot summing only those phenolics with known PPO function (Figure 5) achieves an exceptional 99.1% total variance explanation. This near-total cumulative variance underscores the high discriminatory power of this reduced set of phenolics. The plot shows unambiguous alignment of genotypes with functional PPO (JI64 and JI1794) with PPO substrates and genotypes with nonfunctional PPO (Trendy, JI92) with inhibitors. This tightly defined structure provides clear biochemical insight and is ideal for identifying chemomarkers or selecting breeding lines with targeted PPO phenotypes.^67^ By including only compounds with established roles as PPO substrates or inhibitors, this approach directly targets the enzymatic mechanism under investigation. The exclusion of undefined or ambiguous phenolics minimizes background noise, resulting in a cleaner and more meaningful data set (compared to Figure S4). Moreover, it provides a clear separation between genotypes based on their PPO status, which align distinctly with the profiles of substrates and inhibitors, respectively.

**Figure 5:**
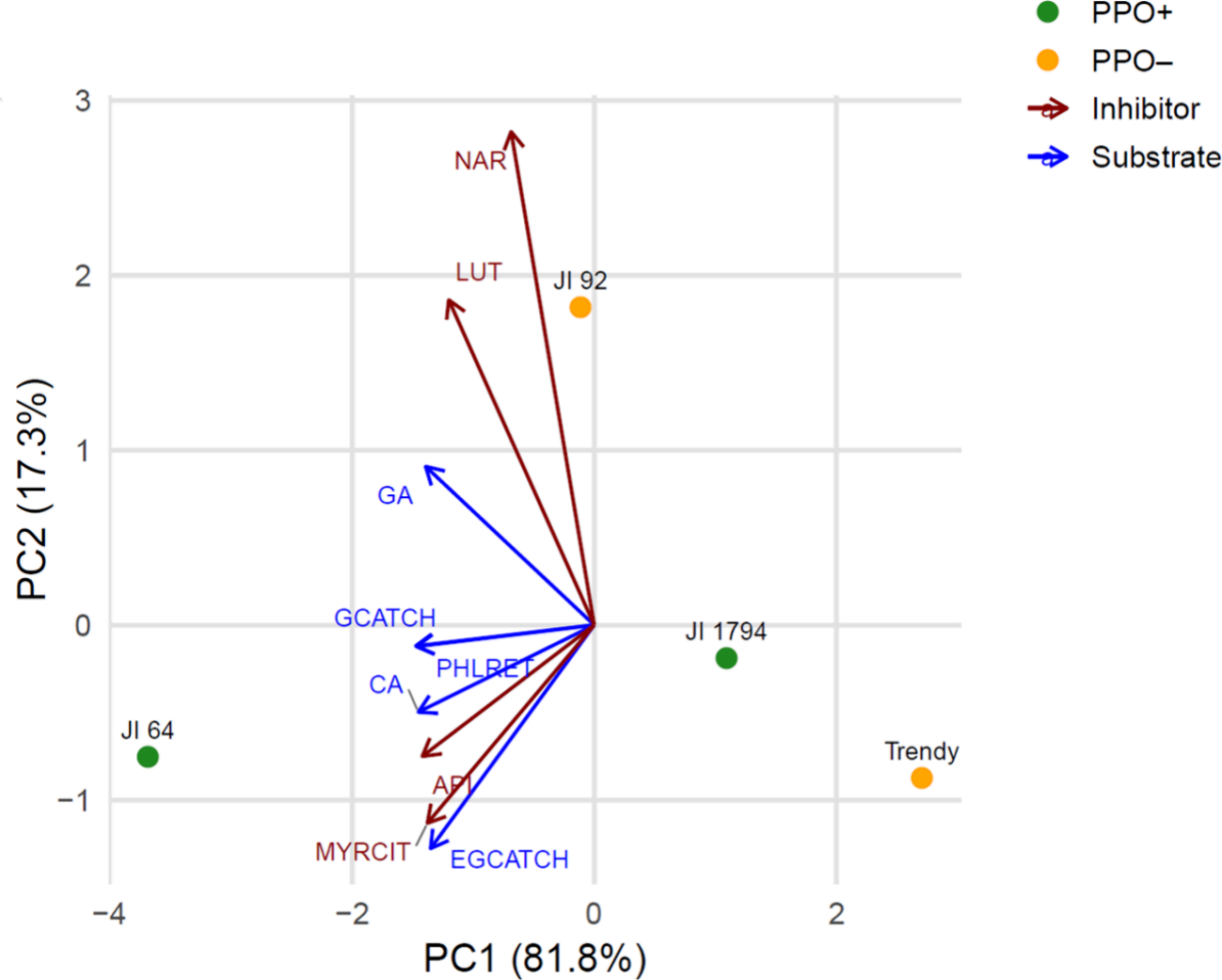
Principal component analysis (PCA) using PPO-relevant phenolics without undefined compounds. Genotypes are color-coded by the PPO status (PPO+, JI1794, JI64, vs ppo–, cv. Trendy, JI92), and phenolics are categorized by known or putative interaction with PPO (substrates, inhibitors). The PCA involving also undefined compounds is shown in Figure S4. Abbreviations: PPO, polyphenol oxidase; GA, gallic acid; 23DHBA, 2,3-dihydroxybenzoic acid; SaAG, salicylic acid glucoside; 4HBA, 4-hydroxybenzoic acid; CA, caffeic acid; pCA, *p*-coumaric acid; GCATCH, gallocatechin; EGCATCH, epigallocatechin; ABI, abietin; MYRCIT, myricitrin; MYRCET; myricetin; PHLRDZ, phloridzin; ERIOD, eriodictyol; LUT, luteolin; API, apigenin; PHLRET, phloretin; NAR, naringenin; CHRYS, chrysin.

### Substrate Specificity of *Pea*PPO

Dopamine and 4-methylcatechol were the best substrates for *Pea*PPO, while the conversion of 2,3-dihydroxybenzoic acid (the main phenolic compound in the wild pea seed coat) and tyramine was catalyzed at a slower rate (Table 2). Besides, several substrates (2,6-dimethoxyphenol, salicylic acid, vitexin, and coniferyl alcohol) were not converted by *Pea*PPO (not shown). Altogether, *Pea*PPO accepted phenols, catechols, and pyrogallols substrates. Thus, *Pea*PPO had both monophenolase and catechol oxidase activity indicating that it can be classified as a tyrosinase.

### Analysis of Products of Reactions Catalyzed by *Pea*PPO

To gain a more detailed understanding of PeaPPO’s interactions with the investigated substrates, the oligomeric products generated by PeaPPO-catalyzed reactions were analyzed using UHPLC-MS, and their structures were proposed based on mass spectral data (Figure 6). Colored products were observed using phloretin, 2,6-dimethoxyphenol, epicatechin, 4-methylcatechol, chlorogenic acid, caffeic acid, pyrogallol, myricetin, and gallocatechin gallate as substrates. Identified oligomers included especially mono-, di-, and trimers (Table 3). Concurrently, *Pea*PPO activity was not confirmed for 2,3-dihydroxybenzoic acid, salicylic acid, and coniferyl alcohol substrates, where only initial substrates were observed (Figure S2). The products of tyramine, dopamine, and L-DOPA conversion by *Pea*PPO were fully precipitated and could not be analyzed.

**Figure 6:**
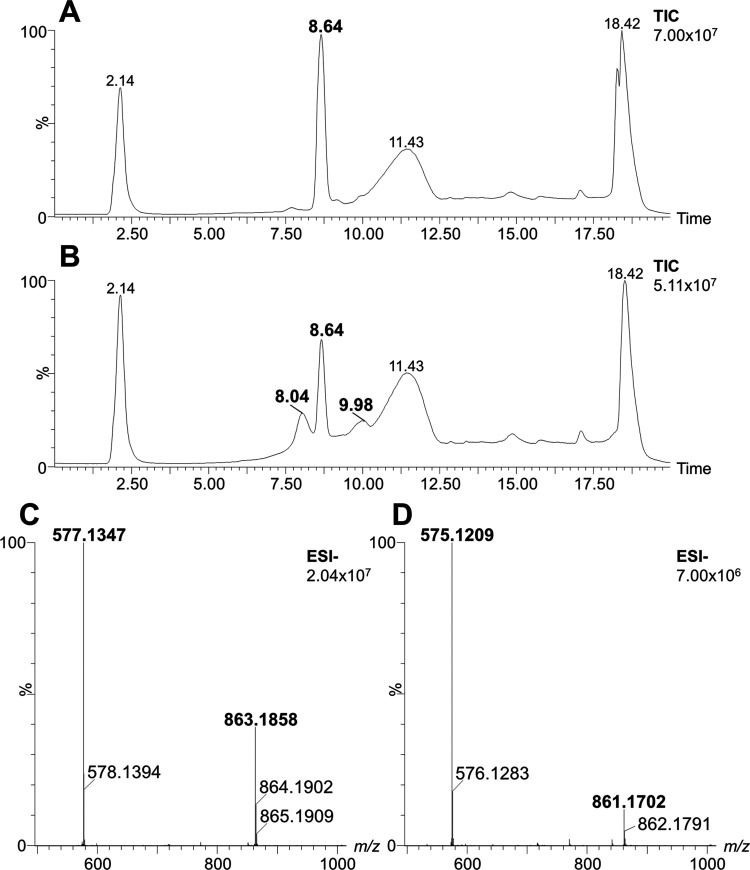
Chromatographic separations of the reaction mixture with epicatechin (Rt 8.64 min, without *Pea*PPO (A), and with *Pea*PPO (B) and related mass spectra combined in retention time ((C) Rt 8.04 min, (D) Rt 9.98 min) with signals of newly formed structures.

**Table 3:** Level of Oligomerization of Studied Substrates after *Pea*PPO Catalysis^a^

substrate (monomer)	oligomers	signal *m*/*z* Δ*m* (ppm)	Rt (min)
epicatechin	dimer	577.1347	8.04
		0.2	
	trimer (−2H)	863.1858	8.04
		4.1	
	dimer (−2H)	575.1209	9.98
		3.3	
	trimer (−4H)	861.1702	9.98
		4.1	
chlorogenic acid	dimer	705.1706	8.48
		5.5	
	trimer (−2H)	1055.2271	8.48
		–3.2	
caffeic acid	dimer	357.0664	9.28
		15.1	
	trimer	535.0896	9.77
		3.6	
	tetramer (+2H)	715.1351	9.77
		7.3	
	pentamer (+2H)	893.1596	10.13
		3.5	
2,6-dimethoxyphenol	dimer	305.0998	10.08
		–8.8	
pyrogallol	tetramer (−2H_2_O)	461.0503	11.62
		–1.3	
4-methylcatechol	heptamer (+O, + H_2_O)	889.2252	9.18
		–10.3	
phloretin	monomer (+2O, −2H)	303.0569	6.22
		21.1	
	dimer (+3O, −2H)	591.1206	9.39
		11.3	
	trimer (+3O, −2H)	863.1915	10.38
		10.7	
	dimer (+O, −2H)	559.1265	11.36
		4.5	
myricetin	dimer (−2H)	631. 0340	9.20
		–3.2	
	trimer (−2H)	947.0569	9.20
		–1.1	
	dimer	633.0511	9.77
		–0.9	
	dimer (+O)	649.0457	9.77
		–1.4	
	trimer	949.0696	9.77
		–4.2	
gallocatechin gallate	dimer	913.1440	7.57
		–2.6	

a Δ*m*: difference from theoretical mass, RT: retention time.

Interestingly, in the case of epicatechin, the control (reaction mixture without *Pea*PPO) contained molecules with a retention time of 8.64 min (Figure 6A), while newly formed structures upon *Pea*PPO catalysis displayed two different retention times (Figure 6B). Epicatechin dimer (*m*/*z* 577.1347) and dehydrogenated epicatechin trimer (*m*/*z* 863.1858) were identified in mass spectra at the retention time of 8.04 min (Figure 6C). Dehydrogenated epicatechin dimer (*m*/*z* 575.1209) and double dehydrogenated epicatechin trimer (*m*/*z* 861.1702) were identified in mass spectra at the retention time of 9.98 min (Figure 6D). The presence of the described molecules was confirmed using MS/MS measurements after the collision-activated dissociation of parent ions in the trap collision cell. Characteristic fragments and appropriate losses were observed in MS/MS spectra, and they are listed in Table S4 for epicatechin oligomers. The products of other substrates after reaction with *Pea*PPO were identified similarly, and their characteristic fragments are listed in Tables S5–S12.

The analyses confirmed the involvement of *Pea*PPO in the formation of colored oligomeric structures. The mutual reactions of substrate monomers and their dehydrogenation or dehydration products are commonly observed. During *Pea*PPO catalysis, the radical formations in the substrate structure increased the occurrence of dehydrogenation and oxidation processes in oligomers.

## Discussion

This work describes the heterologous expression of *Pea*PPO and the determination of its biochemical properties. The PPO gene has been previously identified to be differentially expressed between wild and domesticated pea seeds, with seed-specific expression resulting in the detectable phenotype of hilum region pigmentation.^12^ Legumes, especially their seeds, are rich in polyphenolic substances.^1,68^ Polyphenols constitute a diverse group of secondary metabolites commonly known as antinutritional agents (influencing palatability and digestibility) and as beneficial compounds such as antioxidants or plant defense molecules. Consequently, the phenolic content^10,66,69–71^ and related PPO activity have been extensively studied in legumes.^12,72–74^

In the presence of oxygen, PPOs perform two distinct catalytic reactions: the hydroxylation of phenols to *o*-dihydroxybenzenes (=catechols, monophenolase activity, and tyrosinases only) and the oxidation of catechols to *o*-quinones (catechol oxidase activity, both tyrosinases, and catechol oxidases).^14,17,22,75^ These highly reactive compounds react nonenzymatically through self-polymerizing or covalently bonding and cross-linking with proteins to form high-molecular-weight pigments^26,33^ and have roles in the radical coupling of monolignols to form lignin and flavonoid polymerization in the cell wall.^16,26^ This is likely also the case of *Pea*PPO; however, it remains to be tested.

PPO from numerous plants has been heterologously expressed. These include tomato,^49^ walnut,^76^ olive,^77^ and apricot^64,78^ using the same expression system as applied for *Pea*PPO. For cloning, the *PPO* gene was usually modified for easier expression by removing the signal sequence(s) at the N-terminus (size around 4–9 kDa) that are not directly related to enzymatic activity.^21^ Similarly, we have been unable to generate enzymatically active full-length *Pea*PPO, whereas removal of the N-terminal part rectified this problem (data not shown). This can be caused by the fact that plant signal peptides are usually not recognized by bacteria. Although the whole sequence is translated into a protein, the signal peptides cannot be processed properly by bacteria and may cause the protein to aggregate during expression. The molecular weight of the expressed latent form of *Pea*PPO without the N-terminal signal sequence was 58 kDa according to SDS-PAGE and corresponded to the predicted size. Moreover, we have confirmed the identity of the expressed *Pea*PPO protein by mass spectrometry. Performed MS analysis identified 27 tryptic peptides that covered 64.3% of the sequence of recombinant *Pea*PPO. However, three parts of the sequence were not covered by peptide identification: two near the N-terminus and one at the C-terminus. These can be a result of limitations in trypsin digestion and mass spectrometry. Trypsin predominantly generates short peptides (0.6–3 kDa), leaving extremely short or long peptides underrepresented. Besides, larger or smaller peptides are difficult to ionize, fragment, and analyze, leading to incomplete sequence coverage.^79,80^

In plants, PPOs are translated as latent pro-PPOs composed of the N-terminal transit peptide, the catalytic domain housing two copper ions, followed by a disordered linker and a C-terminal shielding domain.^81^ The import, processing, and localization to plastids were demonstrated for tomato PPO,^82^ and it is also predicted for *Pea*PPO^12^ in agreement with findings that about 75% of PPO proteins possess an N-terminus plastid transit peptide and are predicted to accumulate in the thylakoid lumen.^21^ On the other hand, nonplastidial localization was confirmed for the aureusidin synthase, homologue of polyphenol oxidase, expressed in flowers of snapdragon,^83^ PPO13 from poplar in the vacuolar lumen^84^ and cherimoya PPO localized in the Golgi-network.^85^

A common feature of plant PPOs is the need for activation.^21^ The activity of the latent form of PPO *in vitro* is very low, and it needs to be activated with proteases, acidic pH, fatty acids, or detergents such as SDS. A native *Pea*PPO protein, similarly to PPOs isolated from apricot, olive, walnut, or grape,^48,64,77,86,87^ could be activated by proteolytic cleavage of the shielding domain.^88^ Based on the crystal structures of the native walnut and grape proteins,^86,87^ two highly conserved amino acid residues (PW motif at the end of the active domain of plant PPOs^89^) might signalize for the C-terminal cleavage in the native *Pea*PPO (Figure S3). However, further research must be conducted to characterize the cleavage site of *Pea*PPO. *Pea*PPO was not active in the absence of SDS indicating that it was purified in its latent form. Indeed, *Pea*PPO activity was increased in the presence of 0.25 mM SDS and was markedly decreased at higher concentrations. It has been shown that activation by acidic pH is not as effective as activation of the latent enzyme by the addition of SDS,^90^ which was also the case for *Pea*PPO. The activation of PPO by SDS involves a reorganization of the tertiary structure of the protein^91^ improving the accessibility of the substrates to the active site, without affecting its integrity.^92^ Interestingly, most enzymes are usually inactivated by SDS; however, there are a few exceptions including enzymes such as PPOs or pyruvate oxidases (Russell et al., 1977).^93^ Notably, PPOs from apricot and apple were shown to have the ability to autoactivate by a protease-independent mode.^64,90^ Multiple pH optima of PPO have been described in the literature depending on its substrate (usually catechol or 4-methylcatechol) and plant species, mostly ranging from pH 5.0 to 8.0. The pH optimum of *Pea*PPO was slightly acidic to neutral (5.2–7.0), which is consistent with the pH optima of PPOs from other plants (summarized in Zhang, 2023 ^32^). PPOs were purified and characterized in various legume seeds and sprouts with pH optima ranging between 4 and 7 for the tested substrates like catechol and 4-methylcatechol.^74,94–100^ These largely correspond to our findings on *Pea*PPO. On the other hand, PPO from mushroom has a much wider range of optimal pH (5.0–10.0).^101^

Predicted *Pea*PPO contains two copper atoms in its active site coordinated by three histidine residues each, whereas experimentally, we detected three Cu ions per protein chain. Thus, *Pea*PPO contained one additional copper ion likely localized near the N-terminus of the enzyme at the two conserved disulfide bridges found in many plant PPOs.^59,60^ The valence state and bridging mode of the two copper ions (CuA and CuB) are closely related to the PPO activity, and the PPO is classified into three states based on its interaction with copper and oxygen: met-PPO (Cu^2+^–OH^–^–Cu^2+^), deoxy-PPO (Cu^+^–Cu^+^, no bridging oxygen), and oxy-PPO (Cu^2+^–O_2_^2–^–Cu^2+^). The three states are interconverted in the catalytic process. Met-PPO can only oxidize catechols, while oxy-PPO can catalyze the hydroxylation and oxidation of both phenols and catechols. Deoxy-PPO has no catalytic activity, but it can be converted to oxy-PPO by reaction with oxygen.^38^

Pea seed coat contains a large spectrum of potential substrates for PPO. Comparative metabolomic analysis of wild and cultivated pea seeds showed that colored seeds had significantly higher (6-fold) content of free phenolic acids.^6,8,69,70,102^ Analysis of cultivated and pigmented JI92 compared to wild pea JI64 samples showed differential content of numerous flavonoids and phenolic substances.^6,10^ Targeted analysis of phenolic compounds presented in this study showed a high content of epigallocatechin, 2,3-dihydroxybenzoic acid, and myricitrin (Table S3) and the presence of several previously reported PPO substrates (such as hydroxycinnamic acids). Several phenolic compounds that we identified to be abundant in the pea seed coat (2,3-dihydroxybenzoic acid, myricetin, phloretin, and caffeic acid) were tested as potential substrates of *Pea*PPO since its *in vivo* substrates are still not completely clear.

Depending on whether they accept phenols or only catechols as substrates, PPOs are classified as tyrosinases or catechol oxidases (catecholases). Testing of the pea PPO enzyme with selected substrates showed that *Pea*PPO accepted phenols (tyramine, phloretin), catechols (epicatechin, 4-methylcatechol, dopamine, L-DOPA, chlorogenic acid, and caffeic acid), and pyrogallols (pyrogallol, myricetin, and gallocatechin gallate), indicating its tyrosinase nature. The tested substrates that were processed by *Pea*PPO are in agreement with the substrates listed by Tilley et al., (2023),^23^ although *Pea*PPO showed higher activity toward 4-methylcatechol and dopamine (Table 2). Interestingly, dopamine and L-DOPA were described to be involved in melanin synthesis in insects.^103^ In plants, pigments derived from dopamine are rare and belong to the betalain group (the main pigments of Caryophyllales).^104^ However, recently, it has been assumed that PPO plays a role in L-DOPA and dopamine biosynthesis in plants and a high dopamine content in fava bean seeds has been found.^105^

Following the *Pea*PPO substrate specificity determination, the levels of polymerization of studied substrates (Table 3) after *Pea*PPO catalysis were investigated. The polymerization of 2,3-dihydroxybenzoic acid, salicylic acid, and coniferyl alcohol was not detected, indicating that these phenolics are not converted by *Pea*PPO (Figure S2). The dimers and trimers of studied substrates were the most common oligomers found after *Pea*PPO catalysis. Besides, pyrogallol, caffeic acid, and 4-methylcatechol were also converted to tetramers, pentamers, and heptamers, respectively. Unfortunately, the products of tyramine, dopamine, and L-DOPA catalysis precipitated, and thus, it was not possible to analyze them by the used methods. An appropriate procedure for analysis of the formed precipitates is an object for further research.

Generally, PPO displays high activity on substrates bearing the hydroxyl groups at the *ortho* (2 or 6) position (as in catechol, 4-methylcatechol, and pyrogallol). On the other hand, substrates with hydroxyl groups in the *meta* (3 or 5) or *para* (4) positions of the ring impeded the conversion of corresponding substrates by PPO activity,^97^ which is in agreement with the results obtained for apricot PPO.^64,106^ An amine group in the substrate also negatively affects the rate of its conversion by PPO.^107^

Tyrosinase (cresolase) activity is typically observed in fungal and mammalian PPOs.^14,60^ However, the recent work focused on recombinant PPO from grape and apricot suggests that plant-derived PPOs can exhibit both activities, albeit with tyrosinase activity being substantially lower.^78,108^ In plants, tyrosinase activity has been also shown, for example, for PPO in walnut *Jr*PPO1,^109^ apple,^110^ or pear.^111^ Moreover, plant-derived phenolic compounds can act as PPO inhibitors, via occupation of the PPO active center as substrate analogues, mainly through electrostatic interactions and hydrogen bonding.^112^ Among these are benzoic acid and cinnamic acid hydroxyl derivatives, which are also present in pea seed coat (Klčová et al., 2024,^10^ and this study).

It was confirmed that the most effective inhibitor, which completely prevents the activity of *Pea*PPOs with both phenols and catechols, is tropolone (a competitive inhibitor for mushroom tyrosinase)^113^ and its derivative thujaplicin. Tropolone is structurally analogous to the substrates of PPO as well as being an effective copper chelator. The effect of tropolone on PPO activity agrees with results shown in grapes, where the inhibition was 87.50%^108^ and in field bean seeds.^95^

The study of pea PPO has relevance to the seed phenotype^12,34^ when the PPO activity results in black pigmentation of the hilum that is composed of polymerized phenolic substances including melanin.^33^ There are three types of melanin being recognized: eumelanins, pheomelanins, and allomelanins.^114^ With eumelanins predominant in animals and microorganisms, and some fungi, pheomelanins, are specific to higher animals, mammals, or birds. Both of them are derivatives of tyrosine. Plant and fungal melanins identified so far were devoid of nitrogen and are generically named allomelanin (other melanins).^114^ Although the composition and role of melanin in plants are still enigmatic, they are suggested to be involved in seed defense providing both mechanical strength and protection against microbial decay.^24,25,33,40,41^

The characterization of PPO activity in pea seeds is of great interest to the food industry being associated with the browning of the sprouts,^74^ postharvest seed darkening,^72^ deterioration of cooking quality and food processing.^112^ The results of this study support the view of polyphenol oxidase activity in the oxidation and subsequent polymerization of specific phenolics in the pea seed coat resulting in the synthesis of the dark compounds (putatively melanin) in the hilum region. Altogether, the findings regarding PPO activity in pea seeds have significant implications for food chemistry and agricultural product processing, particularly in improving the quality, shelf life, and marketability of pea-based products.

## Supplementary Material



## Notes

(Figure S1) Mass spectrometric analysis of *Pea*PPO; (Figure S2) chromatographic separations of the reaction mixture with 2,3-dihydroxybenzoic acid; (Figure S3) multiple protein alignment (Clustal Omega) of biochemically characterized PPOs with confirmed tyrosinase or catechol oxidase activity; (Figure S4) comparison of PCA approaches using all forms vs total values of PPO-relevant phenolics with and without undefined compounds; (Table S1) primers for cloning and expression of *Pea*PPO; (Table S2) list of peptides identified by proteomic analysis related to recombinant *Pea*PPO; (Table S3) phenolic composition (pmol/mg DW) of mature pea seed coats; and (Tables S4–S12) list of identified fragments after *Pea*PPO catalysis of epicatechin, chlorogenic acid, caffeic acid, 2,6-dimethoxyphenol, pyrogallol, methylcatechol, phloretin, myricetin, and gallocatechin gallate (PDF)

## Abbreviations

23DHBA: 3-dihydroxybenzoic acid
4HBA: 4-hydroxybenzoic acid
ABI: abietin
API: apigenin
CA: caffeic acid
CHRYS: chrysin
EGCATCH: epigallocatechin
ERIOD: eriodictyol
GA: gallic acid
GCATCH: gallocatechin
GST-HRV3C: glutathione S-transferase tagged Human rhinovirus 3C protease
L-DOPA: l-3,4-dihydroxyphenylalanine
LUT: luteolin
MES: 2-(*N*-morpholino)­ethanesulfonic acid
MYRCET: myricetin
MYRCIT: myricitrin
NAR: naringenin
pCA: *p*-coumaric acid
PeaPPO: polyphenol oxidase from pea (*Pisum elatius*)
PHLRDZ: phloridzin
PHLRET: phloretin
PMSF: phenylmethylsulfonyl fluoride
POX: peroxidases
PPO: polyphenol oxidase
ROS: reactive oxygen species
SaAG: salicylic acid glucoside
SDS: sodium dodecyl sulfate
UHPLC-MS: ultrahigh-performance liquid chromatography–mass spectrometry
