# Supplementary material for: Biochemical Analysis of Recombinant Pea Seed Coat-Specific Polyphenol Oxidase (PeaPPO) in Relation to Various Phenolic Substrates
Source: J Agric Food Chem. 2025 Aug 21;73(35):21754–68. doi: 10.1021/acs.jafc.5c01839 (PMC12412156; doi:10.1021/acs.jafc.5c01839)
Supplement: Supplementary file 1 [file jf5c01839_si_001.pdf]

Supporting Information

Biochemical analysis of recombinant pea seed coat-specific polyphenol oxidase (*Pea*PPO) in relation to various phenolic substrates

Adéla Franková [1], Matthias Pretzler [2], Jana Balarynová [1], Jana Sekaninová [3], Petra Krejčí [4], Petr Bednář [4], Sanja Ćavar Zeljković [5], Vladan Doupovec [3], Mária Škrabišová [3], René Lenobel [6], Marek Petřivalský [3], Annette Rompel [2], Petr Smýkal [1*]

1) Department of Botany, Faculty of Sciences, Palacký University Olomouc, 77900, Czech Republic
2) Universität Wien, Fakultät für Chemie, Institut für Biophysikalische Chemie, Wien, 1090, Austria
3) Department of Biochemistry, Faculty of Sciences, Palacký University Olomouc, 77900, Czech Republic
4) Department of Analytical Chemistry, Faculty of Sciences, Palacký University Olomouc, 77900, Czech Republic
5) Czech Agrifood Research Center, Olomouc, 77900, Czech Republic
6) Czech Advanced Technology and Research Institute (CATRIN), Palacký University, Olomouc, 77900, Czech Republic
7) Laboratory of Growth Regulators, Faculty of Sciences, Palacký University Olomouc, 77900, Czech Republic

Supplementary Information

```
SPISPPDLST CGPPDLPSDA TPPNINCCPP NSTKIIDFKI PSSNQPLRIR QAAHLVNDEY
LAKYKKAIQL MKALPSNDPR SFTQQANIHC AYCDGAYSQA GFPDLDLQVH NSWLFFPFHR
WYLYFHERIL GSLINDPTFA LPFWNYDAPD GMQFPSIYTD SAPPLYDKLR SASHQPPTII
NLDFNDVDGD ASELISNNLT IMYRQVVSNG KTSKLFLGNT YRAGDESDPG PGSVENVPHG
VVHRWSGDDT QPNLENMGTF YAAARDPIFF SHHSNIDRFW SIWKTLGGKR KDFNDKDWLE
SGFLFYDENK NLVRVKVKDC LDSKNLGYVY QDVEIPWLNA KPTPSRTKVQ KKVQVAQGNL
FGIGEAHAAE INEKSTNSRK YVKFPLVLDN VVSAIVKRPK KSRSKKEKEE EEEVLLIDGI
EFEKNIAIKF DVFINDEDDK VIGPGNTEFA GSFVNVPHSS HGNKKKKINS CLRLGLTDLL
EDLDVDGDDS VVVTLVPRCG KGLVKIKNIK IVLED
```

Figure S1. **Mass spectrometric analysis of *Pea*PPO.** Red lines denote peptide sequences identified using the proteomic approach based on in-solution digestion using the protease trypsin followed by analysis by nanocapillary liquid chromatography coupled with tandem mass spectrometry (Tims-TOF Pro 2).

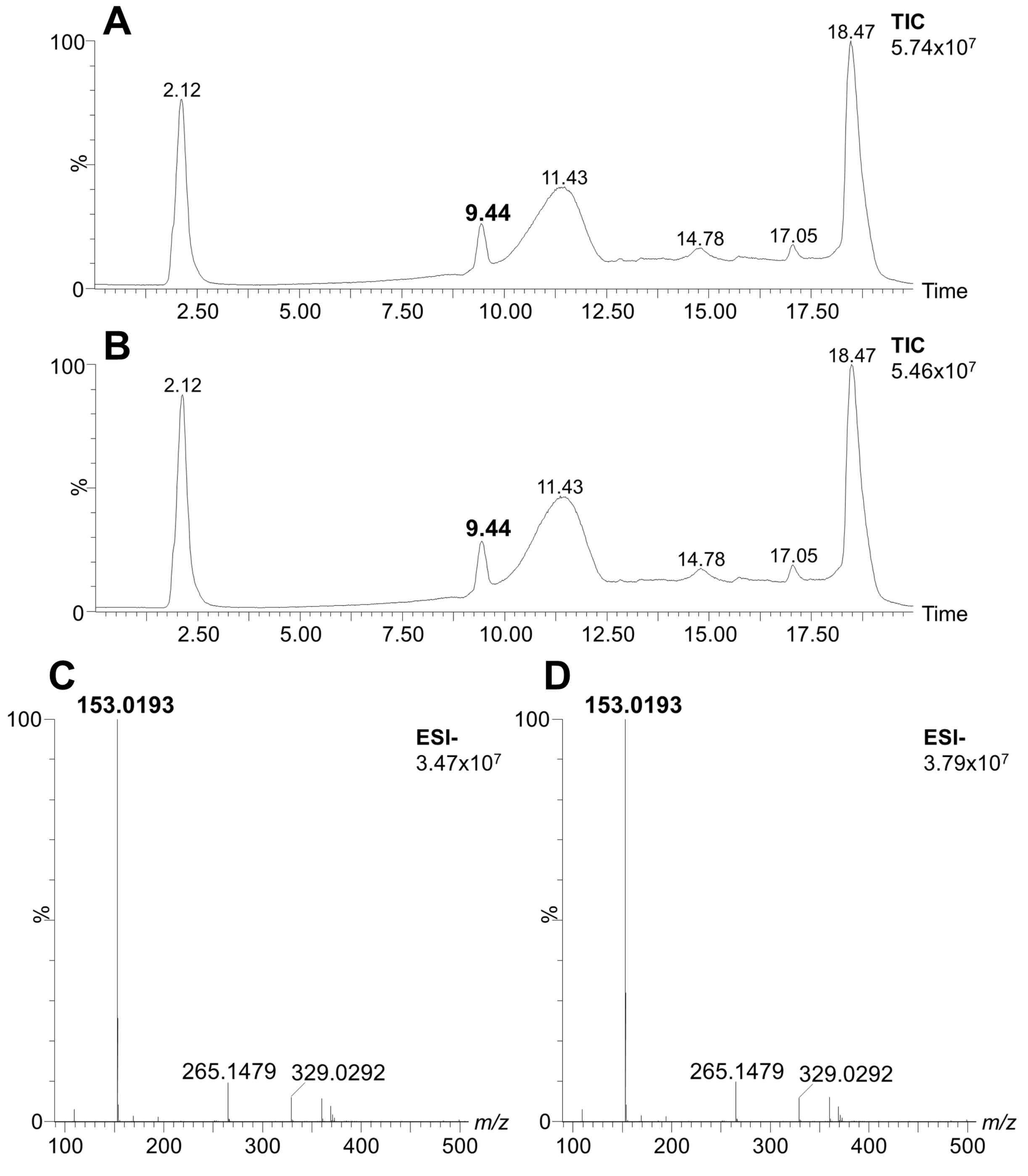

Figure S2. Chromatographic separations of the reaction mixture with 2,3-dihydroxybenzoic acid (Rt 9.44 min), **A**) without presence of *Pea*PPO, **B**) in the presence of *Pea*PPO. Related mass spectra combined in retention time 9.44 min (**C** – from chromatogram A; **D**) from chromatogram B) with signal of 2,3-dihydroxybenzoic acid.

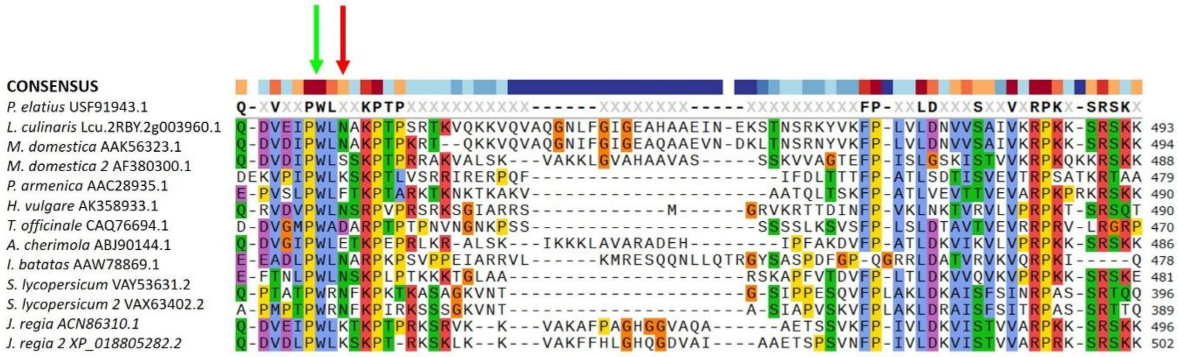

Figure S3. Multiple protein alignment (Clustal Omega) of biochemically characterized PPOs with confirmed tyrosinase or catechol oxidase activity. The alignment shows the C-termini cleavage site (Red arrow) and the two highly conserved amino acid residues (Green arrow) that may signalize for cleavage of the PPO shielding domain based on the crystal structures of native walnut and grape (Bijelic *et al.* 2015; Virador *et al.* 2009) where the C-terminal domain is missing.

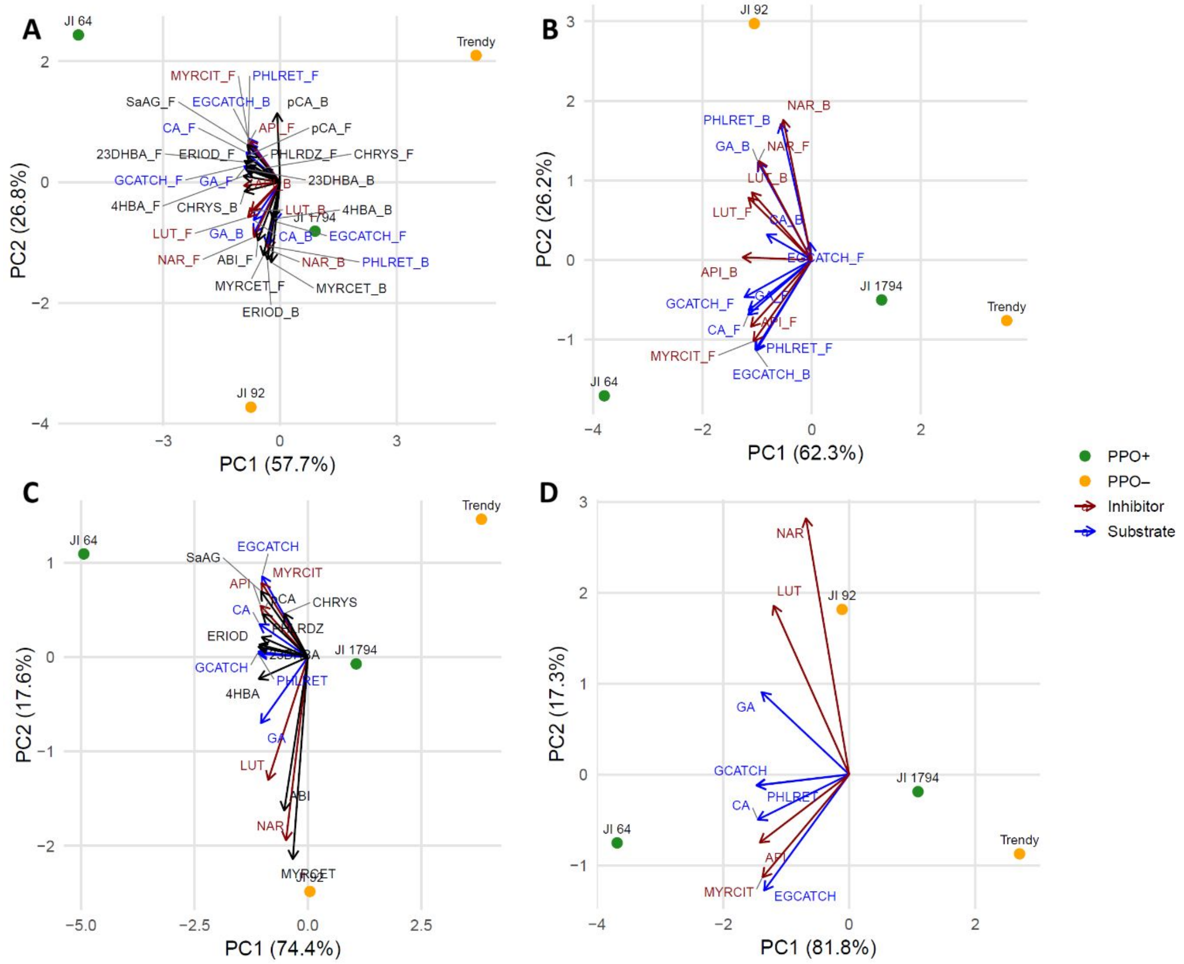

Figure S4. Comparison of PCA approaches using all forms vs. total values of PPO-relevant phenolics with and without undefined compounds. Genotypes are color-coded by PPO status (PPO+, JI1794, JI64, vs ppo–, cv. Trendy, JI92), and phenolics are categorized by known or putative interaction with PPO (substrates, inhibitors, undefined compounds).

The PCA performed using all detected phenolic forms (A) provides a broad overview of chemical diversity in pea seeds. This approach captures both known PPO-related compounds and undefined phenolics, yielding a total variance explanation of 84.5%. The PCA plot reveals a clear separation of genotypes along PC1, indicating that most of the variance is driven by differences in phenolic composition. Genotypes JI1794, JI64, and JI92 are distinctly separated from cv. Trendy, reflecting considerable variation in the levels or ratios of specific phenolic compounds. While this analysis offers a more complete chemical fingerprint, it introduces interpretive ambiguity due to the inclusion of compounds with unclear biological roles. Nonetheless, it effectively distinguishes PPO+ and ppo– genotypes and identifies key compounds associated with enzymatic browning potential, such as gallic acid, caffeic acid, and

phloretin, in alignment with prior PPO studies in legumes (Mayer, 2006; Pourcel *et al*., 2007). Overall, this biplot provides a biologically meaningful discrimination between genotypes based on their phenolic fingerprints and potential PPO reactivity. These results support the idea that phenolic composition is tightly linked to PPO functionality and could guide selection in breeding programs targeting enzymatic browning or antioxidant traits. By contrast, the PCA plot in B retains only phenolic compounds with established substrate or inhibitor function. This refinement improves biological specificity while still explaining 88.5% of the variance. The removal of undefined compounds reduces noise and strengthens genotype separation based on PPO status. Genotypes were clearly separated along these axes, particularly JI64, JI1794, and JI92, which are well distributed in the PCA space. In contrast, cv. Trendy is positioned distinctly along the negative side of PC1, reflecting a divergent phenolic profile compared to the other genotypes. The resulting vector distribution more clearly aligns PPO+ genotypes with known oxidizable substrates, emphasizing a mechanistic link between phenolic composition and PPO activity (Taranto *et al*., 2017). This targeted PCA confirms a biologically coherent pattern between phenolic composition and PPO classification, reinforcing the hypothesis that PPO activity in pea seeds is closely tied to the balance between phenolic substrates and inhibitors. The clear genotype separation also underscores the potential of these phenolics as chemomarkers for selection in breeding programs aimed at modulating enzymatic browning or oxidative resilience. The PCA plot in C integrates all forms of phenolic compounds into their respective totals per compound class. This simplification enables direct comparisons of overall phenolic abundance across genotypes. With 92% of the total variance explained, the analysis preserves strong separation between genotypes, particularly highlighting the divergence of the ppo– genotype (cv. Trendy) from PPO+ lines. However, the inclusion of undefined compounds again introduces interpretive limitations, as their functional significance remains uncertain. While this approach is chemically comprehensive, it may dilute the biological signal relevant to PPO activity. This analysis confirms that PCA of total phenolic levels effectively discriminates genotypes based on both enzymatic activity and compound function.

Abbreviations: PPO, polyphenol oxidase; GA, gallic acid; 23DHBA, 2,3-dihydroxybenzoic acid; SaAG, salicylic acid glucoside; 4HBA, 4-hydroxybenzoic acid; CA, caffeic acid; pCA, *p*-coumaric acid; GCATCH, gallocatechin; EGCATCH, epigallocatechin; ABI, abietin; MYRCIT, myricitrin; MYRCET; myricetin; PHLRDZ, phloridzin; ERIOD, eriodictyol; LUT, luteolin; API, apigenin; PHLRET, phloretin; NAR, naringenin; CHRYS, chrysin; F, free form; B, bound (conjugated) form.

Table S1: **Primers for cloning and expression of *Pea*PPO**

Primer	Sequence 5‘-3‘
*Pea*PPO_EcoRI_fwd	aaacc*GAATTC*ATGGCATCTATCTCACCTC
*Pea*PPO_XhoI_rev	aaacc*CTCGAG*TCAATCTTCAAGCACTATCTTG
l*Pea*PPO_Esp3I_fwd	agct*CGTCTC*caatgTCTCCAATATCTCCACCAGACCTATCC
*Pea*PPO_Esp3I_rev	agct*CGTCTC*atcccTCAATCTTCAAGCACTATCTTGATGTTTTTGATTTTGAC

Table S2

List of peptides identified by proteomic analysis related to recombinant *Pea*PPO.

**Peptide Sequence**	**Start**	**End**	**Peptide Length**
SPISPPDLSTCGPPDLPSDATPPNINCCPPNSTK	1	34	34
IIDFKIPSSNQPLR	35	48	14
IPSSNQPLR	40	48	9
QAAHLVNDEYLAK	51	63	13
QAAHLVNDEYLAKYK	51	65	15
AIQLMKALPSNDPR	67	80	14
WYLYFHER	121	128	8
SASHQPPTIINLDFNDVDGDASELISNNLTIMYR	171	204	34
LFLGNTYR	215	222	8
AGDESDPGPGSVENVPHGVVHR	223	244	22
AGDESDPGPGSVENVPHGVVHRWSGDDTQPNLENMGTFYAAAR	223	265	43
WSGDDTQPNLENMGTFYAAAR	245	265	21
DPIFFSHHSNIDR	266	278	13
FWSIWKTLGGK	279	289	11
DFNDKDWLESGFLFYDENK	292	310	19
DWLESGFLFYDENK	297	310	14
NLGYVYQDVEIPWLNAKPTPSR	325	346	22
KVQVAQGNLFGIGEAHAAEINEK	352	374	23
VQVAQGNLFGIGEAHAAEINEK	353	374	22
YVKFPLVLDNVVSAIVK	381	397	17
FPLVLDNVVSAIVK	384	397	14
FPLVLDNVVSAIVKRPK	384	400	17
EKEEEEEVLLIDGIEFEK	407	424	18
EEEEEVLLIDGIEFEK	409	424	16
FDVFINDEDDK	430	440	11
FDVFINDEDDKVIGPGNTEFAGSFVNVPHSSHGNK	430	464	35
VIGPGNTEFAGSFVNVPHSSHGNK	441	464	24

Identified peptides are sorted according to place in the protein sequence. All identified peptides were confirmed by MS/MS fragmentation by the tandem mass spectrometry analysis with ion mobility separation (TimsTOF Pro2).

Table S3: Phenolic composition (pmol/mg dry weight) of mature pea seed coats.

		Genotype			
**Phenolic compound**	**Form**	**JI64**	**JI1794**	**JI92**	**Trendy**
Gallic acid	Free	104.35 ± 15.43	53.07 ± 5.90	41.03 ± 6.10	1.75 ± 0.39
	Bounded	43.60 ± 10.65	18.08 ± 4.53	64.92 ± 4.90	1.22± 0.72
	Total	147.96 ± 25.6	71.15 ± 5.12	105.94 ± 1.20	2.97 ± 1.10
2,3-Dihydroxybenzoic acid	Free	1557.56 ± 163.08	176.32 ± 11.21	595.79 ± 78.55	5.15 ± 1.02
	Bounded	23.40 ± 1.77	14.99 ± 1.54	9.71 ± 2.65	1.68 ± 0.72
	Total	1580.95 ± 163.50	191.31 ± 11.50	605.50 ± 76.60	6.84 ± 1.54
Salicylic acid glucoside	Free	27.67 ± 4.29	7.48 ± 1.31	3.73 ± 0.73	n.d.
	Bounded	n.d.	n.d.	n.d.	n.d.
	Total	27.67 ± 4.29	7.48 ± 1.31	3.73 ± 0.73	0.00 ± 0.00
4-Hydroxybenzoic acid	Free	296.44 ± 25.66	109.92 ± 5.50	153.53 ± 4.13	6.78 ± 0.67
	Bounded	9.41 ± 1.06	19.92 ± 2.24	10.92 ± 3.20	4.64 ± 0.91
	Total	305.85 ± 26.18	129.84 ± 7.45	164.44 ± 1.52	11.42 ± 0.61
Hydroxybenzoic acids	*Free*	*1986.02 ± 198.98*	*346.80 ± 6.10*	*794.07 ± 69.64*	*13.68 ± 0.77*
	Bounded	*76.41 ± 10.68*	*52.99 ± 7.85*	*85.54 ± 4.38*	*7.54 ± 2.04*
	Total	*2062.42 ± 209.66*	*419.71 ± 7.40*	*879.61 ± 73.80*	*21.22 ± 2.24*
Caffeic acid	Free	33.54 ± 3.42	3.90 ± 0.20	9.23 ± 2.89	0.73 ± 0.08
	Bounded	1.77 ± 0.12	2.03 ± 0.47	1.71 ± 0.33	0.39 ± 0.02
	Total	35.35 ± 3.52	5.93 ± 0.47	10.94 ± 3.01	1.12 ± 0.10
p-Coumaric acid	Free	14.36 ± 1.87	2.13 ± 0.23	6.10 ± 0.73	2.33 ± 0.32
	Bounded	3.19 ± 0.41	1.73 ± 0.19	2.05 ± 0.37	3.13 ± 0.39
	Total	17.55 ± 2.12	3.86 ± 0.21	8.15 ± 0.39	5.45 ± 0.08
Hydroxycinnamic acids	*Free*	*47.04 ± 4.09*	*6.03 ± 0.36*	*15.33 ± 3.57*	*3.06 ± 0.38*
	Bounded	*4.9 ± 0.30*	*3.75 ± 0.33*	*3.76 ± 0.45*	*3.52 ± 0.37*
	Total	*52.90 ± 4.01*	*9.79 ± 0.65*	*19.09 ± 3.30*	*6.57 ± 0.10*
Gallocatechin	Free	444.46 ± 53.72	117.80 ± 24.39	182.36 ± 20.79	0.42 ± 0.12
	Bounded	n.d.	n.d.	n.d.	n.d.

	Total	444.46 ± 53.72	117.80 ± 24.39	182.36 ± 20.79	0.42 ± 0.12
Epigallocatechin	Free	1948.27 ± 215.64	365.09 ± 50.38	94.60 ± 14.29	1.49 ± 0.56
	Bounded	7.54 ± 1.88	27.27 ± 3.05	11.74 ± 1.23	0.96 ± 0.18
	Total	1955.80 ± 216.99	392.36 ± 48.75	106.34 ± 13.20	2.45 ± 0.44
Abietin	Free	19.63 ± 4.29	26.52 ± 1.50	27.87 ± 8.34	n.d.
	Bounded	n.d.	n.d.	n.d.	n.d.
	Total	19.63 ± 4.29	26.52 ± 1.50	27.87 ± 8.34	0.00 ± 0.00
Myricitrin	Free	613.42 ± 52.48	64.39 ± 6.79	50.88 ± 4.79	0.14 ± 0.02
	Bounded	n.d.	n.d.	n.d.	n.d.
	Total	613.42 ± 52.48	64.39 ± 6.79	50.88 ± 4.79	0.14 ± 0.02
Myricetin	Free	15.53 ± 2.01	16.34 ± 0.97	40.24 ± 1.24	0.15 ± 0.03
	Bounded	0.47 ± 0.01	1.22 ± 0.16	1.74 ± 0.32	0.08 ± 0.01
	Total	16.01 ± 2.00	17.56 ± 0.94	41.98 ± 1.37	0.23 ± 0.04
Phloridzin	Free	14.98 ± 1.33	8.78 ± 0.83	5.08 ± 0.44	0.02 ± 0.01
	Bounded	n.d.	n.d.	n.d.	n.d.
	Total	14.98 ± 1.33	8.78 ± 0.83	5.08 ± 0.44	0.02 ± 0.01
Eriodictyol	Free	8.17 ± 0.56	2.37 ± 0.23	3.06 ± 0.06	0.34 ± 0.04
	Bounded	0.12 ± 0.02	0.16 ± 0.05	0.38 ± 0.02	0.02 ± 0.0
	Total	8.29 ± 0.54	2.53 ± 0.27	3.41 ± 0.04	0.36 ± 0.04
Luteolin	Free	376.13 ± 37.64	149.28 ± 12.90	399.91 ± 64.74	1.64 ± 0.28
	Bounded	9.57 ± 1.29	2.38 ± 0.40	10.51 ± 1.69	1.91 ± 0.05
	Total	385.67 ± 38.77	151.67 ± 13.28	410.43 ± 63.05	3.55 ± 0.32
Apigenin	Free	1.49 ± 0.11	0.08 ± 0.00	0.26 ± 0.32	0.02 ± 0.00
	Bounded	0.12 ± 0.02	0.04 ± 0.00	0.08 ± 0.01	0.01 ± 0.00
	Total	1.61 ± 0.09	0.13± 0.01	0.33 ± 0.32	0.03 ± 0.00
Phloretin	Free	0.12 ± 0.02	0.05 ± 0.00	0.02 ± 0.00	0.01 ± 0.00
	Bounded	0.02 ± 0.00	0.01 ± 0.00	0.05 ± 0.01	0.01 ± 0.00
	Total	0.14 ± 0.02	0.06 ± 0.00	0.06 ± 0.01	0.02 ± 0.00
Naringenin	Free	2.22 ± 0.26	1.24 ± 0.08	3.35 ± 0.53	0.06 ± 0.02
	Bounded	0.52 ± 0.05	0.15 ± 0.02	2.26 ± 0.26	0.04 ± 0.00

	Total	2.73 ± 0.29	1.40 ± 0.10	5.60 ± 0.58	0.10 ± 0.02
Chrysoeriol	Free	3.47 ± 0.52	4.77 ± 0.42	0.39 ± 0.10	0.08 ± 0.00
	Bounded	0.10 ± 0.04	0.07 ± 0.02	0.07 ± 0.01	0.02 ± 0.00
	Total	3.57 ± 0.51	4.84 ± 0.41	0.46 ± 0.11	0.10 ± 0.01
Flavonoids	*Free*	*3447.88 ± 169.75*	*756.72 ± 51.34*	*808.01 ± 73.77*	*4.36 ± 0.76*
	Bounded	*18.45 ± 0.66*	*31.31 ± 3.07*	*26.79 ± 0.81*	*3.05 ± 0.17*
	Total	*3466.34 ± 169.82*	*788.02 ± 48.77*	*834.81 ± 73.01*	*7.41 ± 0.68*
Phenolics compounds quantified	*Free*	*5481.84 ± 304.99*	*1617.41 ± 128.77*	*1109.55 ± 50.39*	*21.09 ± 0.65*
	Bounded	*99.81 ± 9.97*	*116.09 ± 3.59*	*88.05 ± 3.59*	*14.11 ± 2.30*
	Total	*5581.66 ± 313.56*	*1733.51 ± 132.12*	*1197.60 ± 41.48*	*35.21 ± 1.66*

n.d. not detected

Table S4. List of identified fragments after *Pea*PPO catalysis of epicatechin.

Oligomers	Signal *(m/z)*	Fragment *(m/z)*	Fragmentation process
dimer	577.1347	559.1187	Loss of H_2O
		533.1417	Combined loss of H_2O and CO
		439.0984	RDA cleavage
		289.0634	Loss of epicatechin
trimer (-2H)	863.1858	725.1417	RDA cleavage
		577.1347	Loss of epicatechin
		533.1417	Combined loss of epicatechin, H_2O and CO
		289.0634	Loss of epicatechin dimer
dimer (-2H)	575.1209	557.1020	Loss of H_2O
		531.1218	Combined loss of H_2O and CO
		449.0791	RDA cleavage
		437.0753	RDA cleavage
		287.0470	Loss of epicatechin
trimer (-4H)	861.1702	723.1247	RDA cleavage
		575.1163	Loss of epicatechin
		531.1183	Combined loss of epicatechin, H_2O and CO
		449.0791	RDA cleavage
		287.0470	Loss of epicatechin dimer

Table S5. List of identified fragments after *Pea*PPO catalysis of chlorogenic acid.

Oligomers	Signal *(m/z)*	Fragment *(m/z)*	Fragmentation process
dimer	705.1706	513.0980	Loss of quinic acid
		351.0708	Loss of chlorogenic acid
		321.0315	Loss of two quinic acid molecules
		191.0514	Loss of chlorogenic and caffeic acid
trimer (-2H)	1055.2271	879.1663	Loss of quinic acid
		705.1706	Loss of chlorogenic acid
		351.0708	Loss of two chlorogenic acid molecules
		191.0514	Loss of two chlorogenic acid molecules and loss of caffeic acid

Table S6. List of identified fragments after *Pea*PPO catalysis of caffeic acid.

Oligomers	Signal *(m/z)*	Fragment *(m/z)*	Fragmentation process
dimer	357.0664	339.0442	Loss of H_2O
		159.0115	Loss of caffeic acid and H_2O
		147.0106	Loss of caffeic acid and CO
trimer	535.0896	357.0664	Loss of caffeic acid
		339.0442	Loss of caffeic acid and H_2O
		179.0297	Loss of two caffeic acid molecules
		159.0115	Loss of two caffeic acid molecules and loss of H_2O
		147.0106	Loss of two caffeic acid molecules and loss of CO
tetramer (+2H)	715.1351	357.0664	Loss of two caffeic acid molecules
		179.0297	Loss of three caffeic acid molecules
		159.0115	Loss of three caffeic acid molecules and loss of H_2O
		147.0106	Loss of three caffeic acid molecules and loss of CO
pentamer (+2H)	893.1596	715.1351	Loss of caffeic acid
		535.0896	Loss of two caffeic acid molecules
		179.0297	Loss of four caffeic acid molecules
		159.0115	Loss of four caffeic acid molecules and loss of H_2O
		147.0106	Loss of four caffeic acid molecules and loss of CO

Table S7. List of identified fragments after *Pea*PPO catalysis of 2,6-dimethoxyphenol.

Oligomer	Signal *(m/z)*	Fragment *(m/z)*	Fragmentation process
dimer	305.0998	289.0696	Loss of CH_4
		273.0689	Loss of CH_3OH
		153.0502	Loss of dimethoxyphenol

Table S8. List of identified fragments after *Pea*PPO catalysis of pyrogallol.

Oligomer	Signal *(m/z)*	Fragment *(m/z)*	Fragmentation process
tetramer (-2H_2O)	461.0503	433.0469	Loss of CO
		219.0317	Loss of two dehydrated pyrogallol molecules and loss of CO

Table S9. List of identified fragments after *Pea*PPO catalysis of methylcatechol.

Oligomer	Signal *(m/z)*	Fragment *(m/z)*	Fragmentation process
heptamer (+O, +H_2O)	889.2252	767.1709	Loss of methylcatechol
		694.1603	Loss of two methylcatechol molecules
		572.1283	Loss of three methylcatechol molecules

Table S10. List of identified fragments after *Pea*PPO catalysis of phloretin.

Oligomers	Signal *(m/z)*	Fragment *(m/z)*	Fragmentation process
monomer (+2O, -2H)	303.0569	259.0573	Loss of H_2O and CO
dimer (+3O, -2H)	591.1206	379.0782	RDA cleavage
		287.0545	Loss of double oxidated phloretin
trimer (+3O, -2H)	863.1915	575.1163	Loss of oxidated phloretin
		287.0545	Loss of two oxidated phloretin molecules
		259.0573	Loss of two oxidated phloretin molecules and loss of H_2O and CO
dimer (+O, -2H)	559.1265	541.1040	Loss of H_2O
		379.0782	RDA cleavage
		287.0545	Loss of oxidated phloretin

Table S11. List of identified fragments after *Pea*PPO catalysis of myricetin.

Oligomers	Signal *(m/z)*	Fragment *(m/z)*	Fragmentation process
dimer (-2H)	631. 0340	315.0176	Loss of myricetin
		451.0333	RDA cleavage
trimer (-2H)	947.0569	631. 0340	Loss of myricetin
		497.0417	RDA cleavage
		315.0176	Loss of two myricetin molecules
dimer	633.0511	315.0176	Loss of myricetin
dimer (+O)	649.0457	631.0340	Loss of H_2O
		523.0201	RDA cleavage
		315.0176	Loss of myricetin and H_2O
trimer	949.0696	631.0340	Loss of myricetin
		497.0417	RDA cleavage

		315.0176	Loss of two myricetin molecules

Table S12. List of identified fragments after *Pea*PPO catalysis of gallocatechin gallate.

Oligomer	Signal *(m/z)*	Fragment *(m/z)*	Fragmentation process
dimer	913.1440	743.1320	Loss of gallate
		455.0638	Loss of gallocatechin gallate
		437.0538	Loss of gallocatechin gallate and H_2O
